# Proximity interactions of the ubiquitin ligase Mind bomb 1 reveal a role in regulation of epithelial polarity complex proteins

**DOI:** 10.1038/s41598-019-48902-x

**Published:** 2019-08-28

**Authors:** Sascha E. Dho, Nancy Silva-Gagliardi, Fabio Morgese, Etienne Coyaud, Emily Lamoureux, Donna M. Berry, Brian Raught, C. Jane McGlade

**Affiliations:** 1 Program in Cell Biology and The Arthur and Sonia Brain Tumour Research Centre, The Hospital for Sick Children, 555 University Ave., Toronto, ON M5G 1X8 Canada; 2 Princess Margaret Cancer Centre, University Health Network, Toronto, ON M5G 1L7 Canada; 3 Department of Medical Biophysics, University of Toronto, Toronto, Canada

**Keywords:** Protein-protein interaction networks, Apicobasal polarity, Apicobasal polarity, Ubiquitylation, Ubiquitylation

## Abstract

MIB1 belongs to the RING domain containing family of E3 ubiquitin ligases. In vertebrates, MIB1 plays an essential role in activation of Notch signaling during development, through the ubiquitination and endocytosis of Notch ligands. More recently, Notch independent functions for MIB1 have been described in centriole homeostasis, dendritic spine outgrowth and directional cell migration. Here we use proximity-dependent biotin identification (BioID) to define the MIB1 interactome that included 163 high confidence interactions with polypeptides linked to centrosomes and cilia, endosomal trafficking, RNA and DNA processing, the ubiquitin system, and cell adhesion. Biochemical analysis identified several proteins within these groups including CCDC14 and EPS15 that were ubiquitinated but not degraded when co-expressed with MIB1. The MIB1 interactome included the epithelial cell polarity protein, EPB41L5. MIB1 binds to and ubiquitinates EPB41L5 resulting in its degradation. Furthermore, MIB1 ubiquitinates the EPB41L5-associated polarity protein CRB1, an important determinant of the apical membrane. In polarized cells, MIB1 localized to the lateral membrane with EPB41L5 and to the tight junction with CRB1, CRB3 and ZO1. Furthermore, over expression of MIB1 resulted in altered epithelial cell morphology and apical membrane expansion. These results support a role for MIB1 in regulation of polarized epithelial cell morphology.

## Introduction

MIB1 belongs to the RING domain-containing family of E3 ligases. It is a multi-domain protein that includes an amino terminal MZM region composed of two regions of homology to the HERC2 ubiquitin ligase (MIB/HERC domains) flanking a ZZ type zinc finger, two adjacent MIB homology repeats (REP), and a central region containing Ankyrin repeats^1–3^. The amino terminal region is required for substrate recognition and in the case of the Notch ligand, Jagged, involves a bipartite recognition mechanism with MZM and REP regions binding to distinct substrate motifs^4^. The carboxy-terminal region of MIB1 includes three RING finger domains with a coiled-coil region (CC) in between RING2 and RING3. Only RING3 is required for the ubiquitination of MIB1 substrates that have been characterized to date; the functions of RING1 and RING2 are unknown^5^.

MIB1 has a conserved function in the activation of Notch signaling during development through the ubiquitination and endocytosis of Notch ligands^2,6–8^. This event is essential for Notch pathway activation where internalization of ligands is thought to create a pulling force on Notch that relieves autoinhibition allowing subsequent downstream cleavage events and the release of Notch intracellular domain^9,10^. In *Drosophila*, both MIB1 and another structurally distinct RING domain E3, Neuralized, play overlapping and discrete roles in the regulation of Notch ligand endocytosis while in vertebrates, only MIB1 appears to be required for Notch signalling^8^.

In mammalian cells, additional roles for MIB1 have also been described. MIB1 was first identified as an interactor of death associated protein kinase (DAPK)^1^, and was proposed to regulate apoptosis through ubiquitination and degradation of DAPK. More recently, MIB1 has been localized to centriolar satellites^11–13^ where it ubiquitinates components including PCM1, AZI1 and TALPID3 and is involved in regulating ciliogenesis^14,15^. MIB1 has also been implicated in centriole duplication through degradation of PLK4^16^. Finally, MIB1 activity is involved in cell migration through ubiquitin-mediated degradation of p120 catenin and the resulting attenuation of Rac1 activity^17^, neuronal dendritic spine outgrowth through CDKL5 ubiquitination^18^, and autophagy through ubiquitination of the ATG8 protein GABARAP^19^.

Thus, in addition to the conserved and well-defined role in the regulation of Notch pathway activation, the identification of functions unrelated to Notch pathway activation reveal that in mammalian cells MIB1 regulates a broad range of cellular functions. Here we describe the MIB1 proximity interactome, providing a comprehensive view of MIB1 interactions in distinct cellular functions, and identify novel MIB1 substrates and interactions related to previously described functions. We also identify interactions with the polarity proteins EPB41L5 and Crumbs and describe a novel role for MIB1 in regulating in the maintenance of epithelial cell shape and morphology.

## Results

### BioID identification of the Mind Bomb 1 proximity interactome

**Figure 1:**
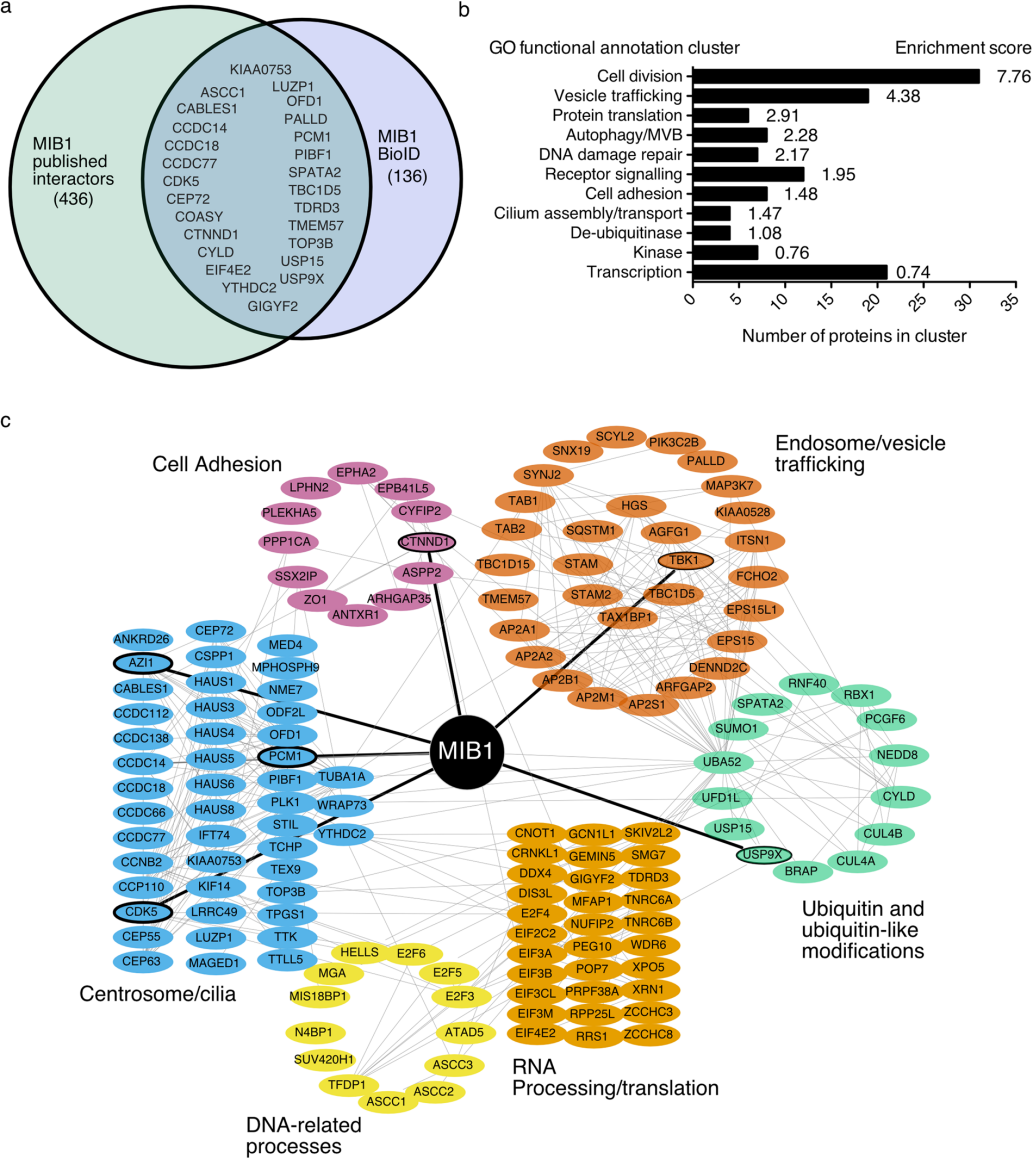
Identification of MIB1 proximity interactome using BioID. (**a**) Venn diagram of the MIB1 BioID protein data set and published MIB1 interactors obtained from BioGRID (https://thebiogrid.org) where MIB1 was bait. (**b**) Graph showing GO functional annotation clusters for the MIB1 BioID dataset. MIB1 proximity interactors were uploaded to the online Database for Annotation, Visualization and Integrated Discovery, DAVID, and clustered using an EASE of 1.0 and medium stringency. (**c**) Schematic representation of 147 of 163 proteins identified using BioID with FlagBirA*-MIB1 expressed in HEK293 cells. SAINT data were uploaded to string-dp.org for network analysis. The resulting network was imported into Cytoscape 3.6.1 for figure preparation. Nodes represent unique proteins identified and categorized according to the indicated function based on information in Entrez Gene, UniProt and primary literature. Light grey edges represent protein-protein interactions determined by STRING (interaction score of 0.4 or greater). Nodes and edges in bold are previously published MIB1 interactors or substrates. The complete list of unique interactors is shown in Table S1.

To identify the human MIB1 proximity interactome, a BirA (R118G) biotin ligase-MIB1 fusion protein was expressed in HEK293 cells in the presence of excess biotin and purified biotinylated protein species identified by LC-MS/MS (Supplementary Table S1). This approach identified 163 unique proteins as part of the MIB1 interactome and included known MIB1 interacting proteins and previously characterized substrates (PCM1, AZI1, USP9x, CDK5, TBK1, CTTND1) (Fig. 1 and Supplementary Table S1). We compared the MIB1 BioID dataset with a list of previously identified MIB1 interactors (determined by affinity purification/mass spectroscopy, Yeast 2-hybrid screen, or affinity purification/Western, where MIB1 was bait), obtained from the curated repository of protein interaction datasets, BioGRID (https://thebiogrid.org). Our screen confirmed 26 interactors from this list, and identified 136 new interactions (Fig. 1a). Proteins identified by MIB1 BioID were clustered based on GO annotations using the Database for Annotation, Visualization and Integrated Discovery, DAVID^20,21^ (Fig. 1b; Supplementary Table S2). Eleven functional clusters were identified that include: cell division, vesicle trafficking, DNA repair, protein translation, signalling, and cell adhesion. Based on analysis of additional information obtained from UniProt, Entrez Gene, and published data, MIB1 proximity interactome was further classified into the following functional clusters: cilia/centrosome, vesicle trafficking, DNA-related processes, RNA processing/translation, and ubiquitin/ubiquitin-like modifications (Fig. 1c).

### Characterization of potential MIB1 substrates identified by BioID

We selected several of the novel MIB1 proximity interactors for further characterization as potential MIB1 ubiquitin ligase substrates based on annotated functions and relationship to known MIB1 interactors. For each putative substrate we assessed whether MIB1 caused either degradation or shift in protein mobility, indicative of possible ubiquitination. Epitope tagged cDNAs encoding selected MIB1 interactors were co-transfected into HEK293T cells with MIB1 or a MIB1 mutant that no longer retains ubiquitin ligase function, MIB1CA, containing a Cys to Ala substitution in the third RING FINGER domain (CA), or MIB1Δ3R, in which all 3 RING domains are deleted.

**Figure 2:**
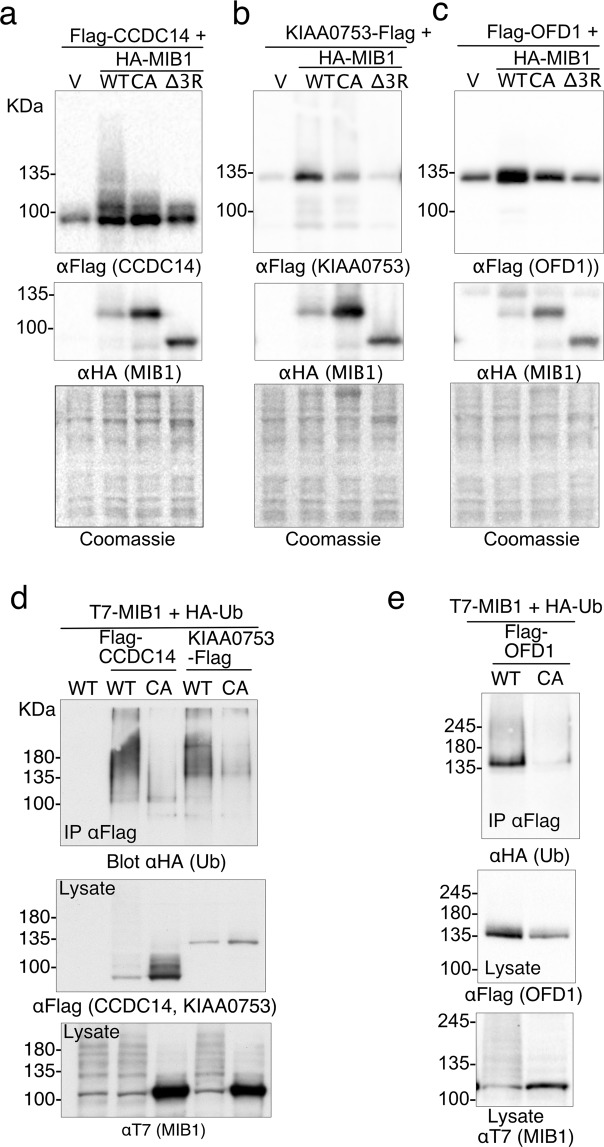
The centrosome-associated proteins CCDC14, KIAA0753 and OFD1 are ubiquitinated when co-expressed with MIB1. (**a**–**c**) Flag-CCDC14, KIAA0753-Flag or Flag-OFD1 were individually co-transfected into HEK293T cells with HA-MIB1 WT, a MIB1 mutant that disrupts the last RING finger domain (CA), or a MIB1 mutant that has the 3 RING domains deleted (Δ3R). After 18 hours, cell proteins were extracted in 1xLaemmli lysis buffer and boiled. Equal concentrations of protein were separated by SDS-PAGE and immunoblotted with either anti-FlagM2 or anti-HA (for MIB1). (**d**,**e**) Flag-tagged CCDC14, KIAA0753 and OFD1 were individually co-transfected with HA-Ub and either T7-MIB1 or T7-MIB1CA mutant. At 18 hours, cells were treated for 4 hours with 10 μM MG132, followed by lysis is 1%SDS/RIPA lysis buffer and boiled. Flag-tagged proteins were immunoprecipitated from an equal concentration of cell lysate, and immunoblotted with anti-HA (Roche) to identify ubiquitinated proteins. Lysates (50 μg) were immunoblotted for Flag-tagged substrate (middle panels) and T7-MIB1 (lower panels). Each figure is representative of at least 3 independent experiments. For clarity some blots were cropped. Full-length blots are shown in Fig. S4.

A number of studies have localized MIB1 to pericentriolar satellites, both through interactions with known satellite proteins (for example, PCM1, AZI1, and CPAP), and through single cell localization of overexpressed and endogenous MIB1^11–14,16^. Functionally, MIB1 has been implicated in centrosomal duplication^16^, ciliogenesis^14,15^, and the ubiquitination of PCM1, AZI1 and TALPID3^14,15^. Two proteins identified in our proximity screen, CCDC14 and KIAA0753, are regulators of centriole duplication with opposing functions, in part through their involvement in regulating the localization of CEP63 to the pericentriolar satellite^11^. Although not directly confirmed, both of these proteins were also identified as putative MIB1 interactors in a high-throughput AP-MS screen^22^. A marked laddering of CCDC14 at higher molecular weights was observed when co-expressed with wild type MIB1 but not with mutants HA-MIB1CA or HA-MIB1Δ3R (Fig. 2a). The effect of MIB1 coexpression on KIAA0753 protein was not as pronounced, although weak laddering was observed, which was decreased with MIB1CA, and abolished with MIB1Δ3R (Fig. 2b). Neither CCDC14 nor KIAA0753 appeared to be degraded in the presence of wild type MIB1, and KIAA0753 protein abundance appeared increased. To assess substrate ubiquitination, cells were also transfected with HA-ubiquitin and putative substrates were immunoprecipitated and then immunoblotted with anti-HA to detect ubiquitinated species. Cell lysates used for the HA-ubiquitination assay were boiled in 1% SDS-containing lysis buffer to dissociate all protein-protein interactions, and thus ensure that the HA-Ub signal observed could be directly attributed to the immunoprecipitated protein of interest. Both CCDC14 and KIAA0753 were ubiquitinated in the presence of HA-ubiquitin and T7-MIB1 but not the MIB1CA mutant (Fig. 2d) as indicated by a high mobility smear detected by both anti-HA and anti-FLAG immunoblot.

OFD1 is a proximity interactor of both CCDC14 and KIAA075^11^ and is reported to form a complex with KIAA0753 at pericentriolar satellites^23^. It localizes to both centriolar satellites^23–25^ and centrioles and is functionally linked to primary cilia formation^25–27^. When co-expressed with HA-MIB1 we observed an increase in OFD1, a proportion of which ran at a slightly higher molecular mass. This did not occur with either MIB1 mutant (Fig. 2c). When Flag-OFD1 was co-expressed with T7-MIB1WT and HA-Ub, a marked HA-Ub signal was seen at the molecular weight of immunoprecipitated Flag-OFD1, and also a weak smear above (Fig. 2e). These data suggest that MIB1 promotes the ubiquitination of CCDC14, KIAA0753 and OFD1 but that this does not lead to degradation of these proteins.

**Figure 3:**
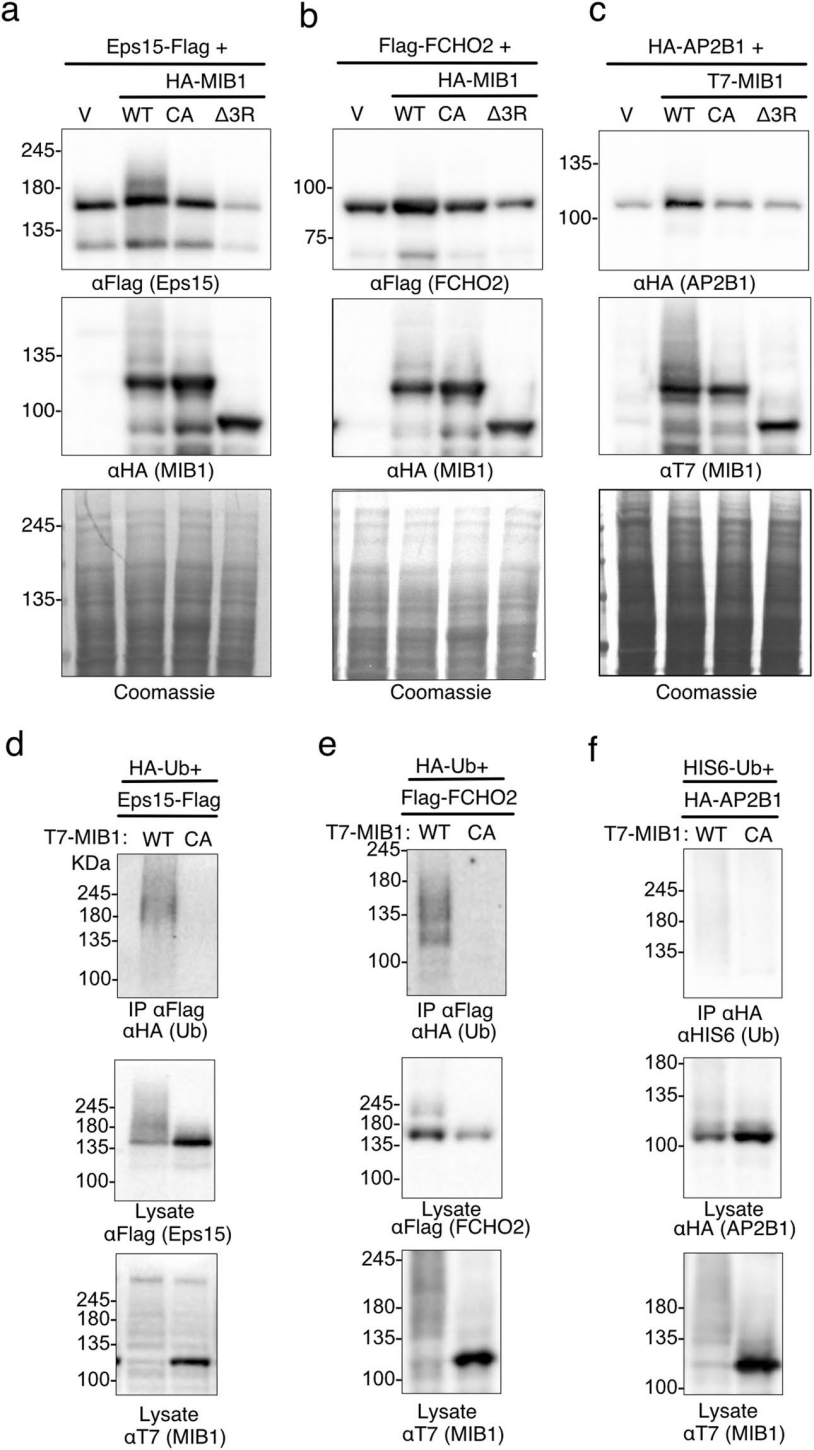
The endocytic proteins EPS15 and FCHO2 are ubiquitinated when co-expressed with MIB1. (**a**–**c**) Eps15-Flag, Flag-FCHO2 or HA-AP2B1 were individually co-transfected into HEK293T cells with either HA-tagged MIB1 WT, or mutants MIB-CA or MIBΔ3 R (Eps15 and FCHO2 samples) or T7-tagged MIB WT and mutants (AP2B1 experiment). After 18 hours, cell proteins were extracted in 1xLaemmli lysis buffer and boiled. Equal concentrations of protein were separated by SDS-PAGE and immunoblotted with either anti-FlagM2, anti-HA (for MIB1 or AP2B1) or anti-T7 (MIB1). (**d**–**f**) Eps15-Flag, Flag-FCHO2, or HA-AP2B1 were co-transfected with T7-MIB1 or T7-MIB1CA mutant, and either HA-Ub (Eps15 and FCHO2) or HIS6-Ub (AP2B1). At 18 hours, cells were treated for 4 hours with 10 μM MG132, followed by lysis in 1%SDS/RIPA lysis buffer and boiled. Tagged substrates were were immunoprecipitated from 2 mg of cell lysate as indicated, and immunoblotted with either anti-HA (Roche) or anti-HIStag to identify ubiquitinated proteins. Lysates (50 μg) were immunoblotted to confirm substrate and T7-MIB1 expression. Each figure is representative of 3 (Eps15), 2 (FCHO2) and 1 (AP2B1) independent experiments. For clarity some blots were cropped. Full length blots are shown in Supplementary Fig. S4.

MIB1 is required for activation of Notch signalling through its role in the ubiquitination and endocytosis of Delta^2,8^. Epsin, an endocytic clathrin-adaptor protein, binds Delta through its ubiquitin interaction motif (UIM) and in turn links it to clathrin directly and/or via other endocytic Epsin-binding proteins^9,28–30^. Although less well studied in the context of Notch ligand endocytosis, ubiquitination of components of the endocytic machinery also regulates membrane protein trafficking^31–33^. MIB1 BioID identified endocytic proteins involved in the formation of clathrin-coated vesicles, including Eps15, FCHO2 and AP2B1, which associate at plasma membrane clathrin coated structures^34,35^. We tested whether these three proteins are also targeted for ubiquitination by MIB1. Expression of HA-MIB1 with Eps15-Flag resulted in detection of a modified Eps15 protein band running just above the unmodified protein (Fig. 3a). When co-expressed with HA-Ub, Eps15-Flag was ubiquitinated by T7-MIB1WT, but not the ligase-dead mutant (Fig. 3d). While MIB1 co-expression did not result in degradation or a notable change the migration of either FCHO2 or AP2B1 (Fig. 3b,c), FCHO2 but not AP2B1 was ubiquitinated in the presence of wild type but not the MIB1-CA mutant (Fig. 3f,g).

**Figure 4:**
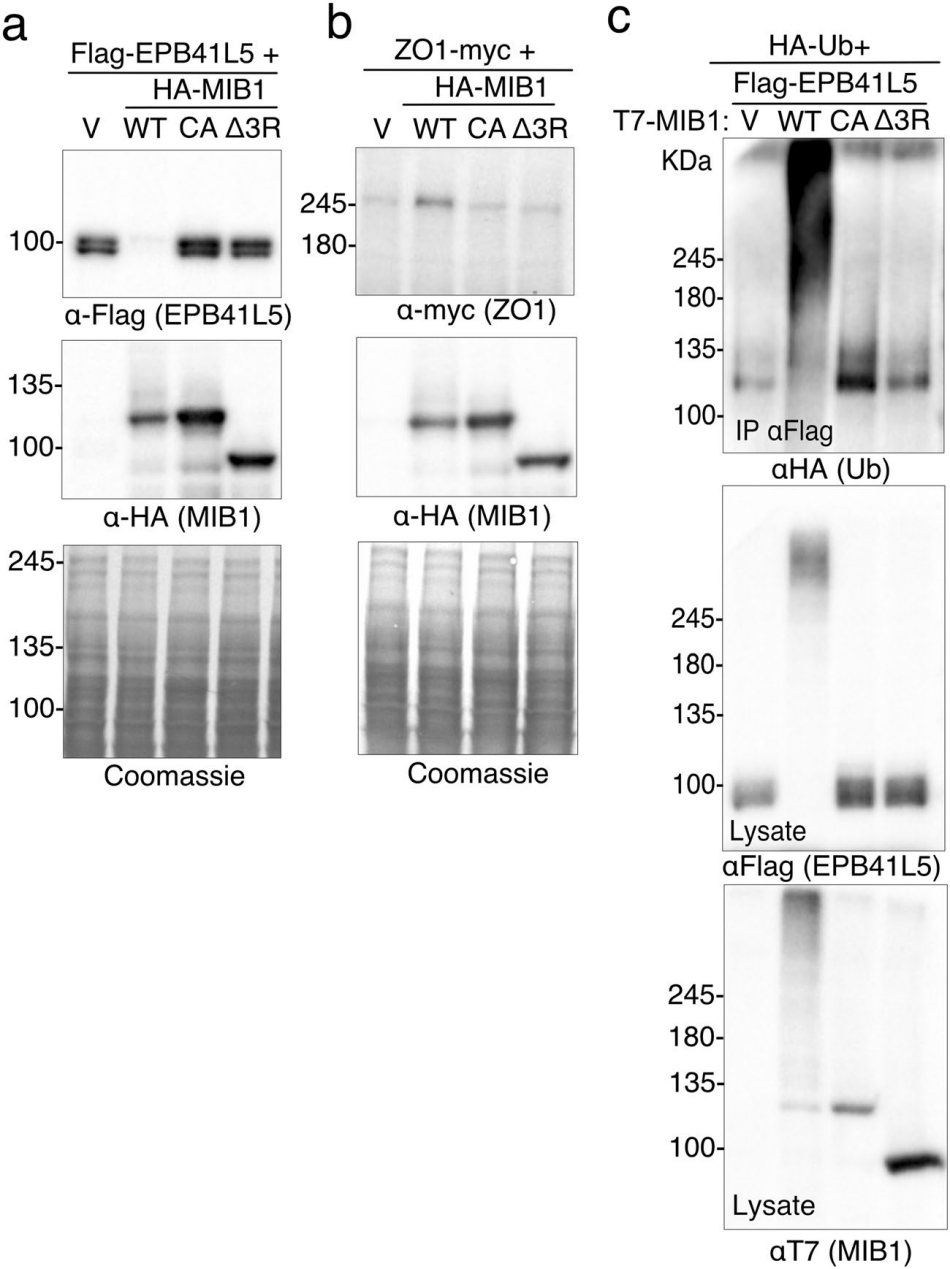
MIB1-mediates ubiquitination and degradation of the polarity protein EPB41L5. (**a**,**b**) HEK293T cells were transiently co-transfected with FLAG-EPB41L5 or ZO1-myc, and HA-MIB1 WT or RING mutant (HA-MIB1CA or HAMIB1Δ3R). Eighteen hours following transfection, 50 μg of cell lysates were resolved by SDS-PAGE and immunoblotted with anti-FlagM2 (**a**, EPB41L5), anti-myc (**b**, ZO1), or anti-HA (MIB1). (**c**) EPB41L5 is ubiquitinated by MIB1. HEK293T cells were transiently co-transfected with Flag-EPB41L5, HA-ubiquitin and either T7-MIB1WT, T7-MIB1CA, or T7-MIB1Δ3R. Eighteen hours post-transfection, cells were treated for 4 hours with 10μM MG132, and cell lysates prepared in 1%SDS/RIPA lysis buffer. Flag-EPB41L5 was immunoprecipitated from 2 mg of the protein lysate with anti-FlagM2 and immunoblotted with anti-HA to detect ubiquitinated proteins (Top). Lysates (50μg) were probed with anti-FlagM2 (middle) and anti-T7 (bottom panel) to confirm expression of EPB41L5 and MIB1. Each figure is representative of at least 3 independent experiments. For clarity some blots were cropped. Full length blots are shown in Supplementary Fig. S4.

We also examined the effects of MIB1 on the membrane associated polarity proteins EPB41L5 and ZO1 (TJP1). Co-transfection of Flag-EPB41L5 with HA-MIB1WT lead to a marked loss of Flag-EPB41L5 protein detected in cell lysates by immunoblotting. Coexpression with the ubiquitin ligase defective mutants MIB1CA or MIB1Δ3R did not result in EPB41L5 degradation (Fig. 4a). When Flag-EPB41L5 and T7-MIB1WT were co-expressed with HA-tagged ubiquitin, immunoprecipitated EPB41L5 was clearly modified by HA-Ubiquitin (Fig. 4c). A high molecular weight smear indicative of EPB41L5 poly-ubiquitination was detected in cells transfected with wild type MIB1 but not MIB1CA or MIB1Δ3R, indicating that in HEK293T cells MIB1 expression causes ubiquitin-mediated degradation of EPB41L5. In contrast, when ZO1-myc was co-expressed with HA-MIB1WT, no shift in molecular weight or degradation of ZO-1 was apparent (Fig. 4b).

### MIB1 prevents EPB41L5-mediated changes in cell morphology

**Figure 5:**
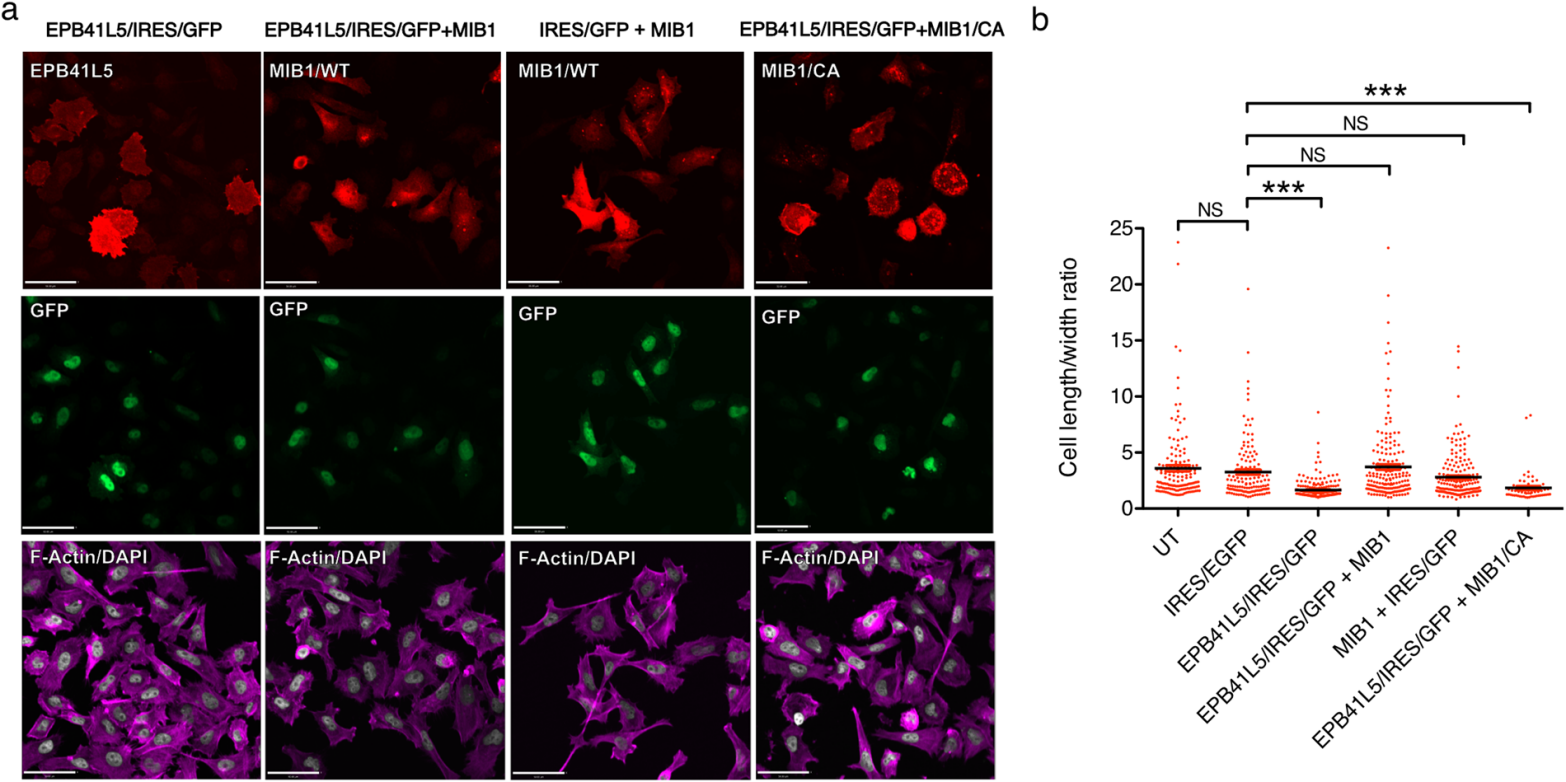
Co-expression of MIB1 prevents EPB41L5 mediated cell shape changes. (**a**) HeLa cells were transiently transfected with (images left to right): Flag-EPB41L5/pIRES/EGFP plus T7/pcDNA3.1, Flag-EPB41L5/IRES/EGFP plus T7-MIB1, T7-MIB1 with empty pIRES2/EGFP, or Flag-EPB41L5/IRES/GFP plus T7-MIB1 CA mutant. After 18 hours cells were trypsinized and reseeded onto fibronectin coated coverslips and allowed to adhere for 2 hours. Cells were fixed, permeablized, and immunostained with anti-FlagM2 (EPB41L5; CY3 anti-mouse, upper row, left), or anti-MIB1 (MIB1; CY3 anti-guinea pig, upper row, 3 right images), anti-GFP (co-expressed GFP; AlexaFluor 488 anti-rabbit, middle row), and Alexa Fluor 635 phalloidin and DAPI (F-actin and nuclei; lower row). Images were acquired at 20X magnification on a Quorum Spinning Confocal microscope and analysed using Perkin Elmer Volocity software. Scale bar is 50 μm. Representative immunoblots showing protein expression are shown in Fig. S2. (**b**) Quantification of cell shape. The cell length to width ratio for each condition was measured: untransfected (UT: 152 cells over 4 experiments), empty IRES2/EGFP and T7/pcDNA3.1 vectors (137 cells over 3 experiments), EPB41L5/IRES/EGFP (219 cells over 4 experiments), EPB41L5/IRES/EGFP plus T7-MIB1 (186 cells over 4 experiments), IRES/EGFP plus T7-MIB1 (184 cells over 4 experiments) and EPB41L5/IRES/EGFP plus T7-MIB1 CA mutant (60 cells, 1 experiment). The summary graph shows individual cell measurements with their mean +/−SEM. for each condition. Statistical significance was measured using a One-Way ANOVA (Kruskal-Wallis) plus Dunn’s Multiple Comparisons test, p < 0.05.

Since EPB41L5 exhibited clear MIB1-dependent degradation we further characterized this interaction. Previously, EPB41L5 overexpression in HeLa cells was reported to cause accumulation of cortical actin and changes in cell morphology^36^. In order to confirm a functional interaction between EPB41L5 and MIB1, we overexpressed EPB41L5 alone in Hela cells and compared the resulting cell morphology to that of cells that were transfected with both EPB41L5 and MIB1 (Fig. 5). As co-expression of EPB41L5 and MIB1 in HeLa cells resulted in a marked loss of EPB41L5 protein expression, Flag-EPB41L5 was expressed from a bicistronic IRES-GFP vector and GFP expression used to identify cells transfected with Flag-EPB41L5 (Supplementary Fig. S1). Cells co-expressing Flag-EPB41L5 and T7-Mib were identified by immunostaining with anti-MIB1 and anti-GFP. As expected, cells expressing Flag-EPB41L5/IRES/EGFP displayed altered morphology and were more circular and spread compared to untransfected cells and cells transfected with IRES/EGFP alone (Fig. 5a). In contrast, the morphology of cells co-transfected with both Flag-EPB41L5/IRES/EGFP and T7-MIB1 reverted to the elongated morphology of cells expressing MIB1 or vector alone control (IRES/EGFP). In contrast, cells transfected with Flag-EPB41L5/IRES/GFP and the MIB1-CA mutant which does not degrade EPB41L5, displayed the altered morphology of cells expressing EPB41L5/IRES/GFP alone. To quantify changes in cell morphology, we calculated the length to width ratio of transfected cells, as a measure of how circular or elongated the cells were (Fig. 5b). The length to width ratio of EPB41L5 expressing cells was significantly different than cells expressing GFP alone or cells in which EBP41L5 was co-expressed with MIB1. Consistent with the observation that MIB1 expression results in E3 ligase-dependent degradation of EPB41L5, cells with co-expression with the MIB1CA mutant had similar length to width ratios to cells expressing EPB41L5 alone, and were significantly different to control cells (Fig. 5b).

### EPB41L5 interacts with the MZM substrate binding region of MIB1

**Figure 6:**
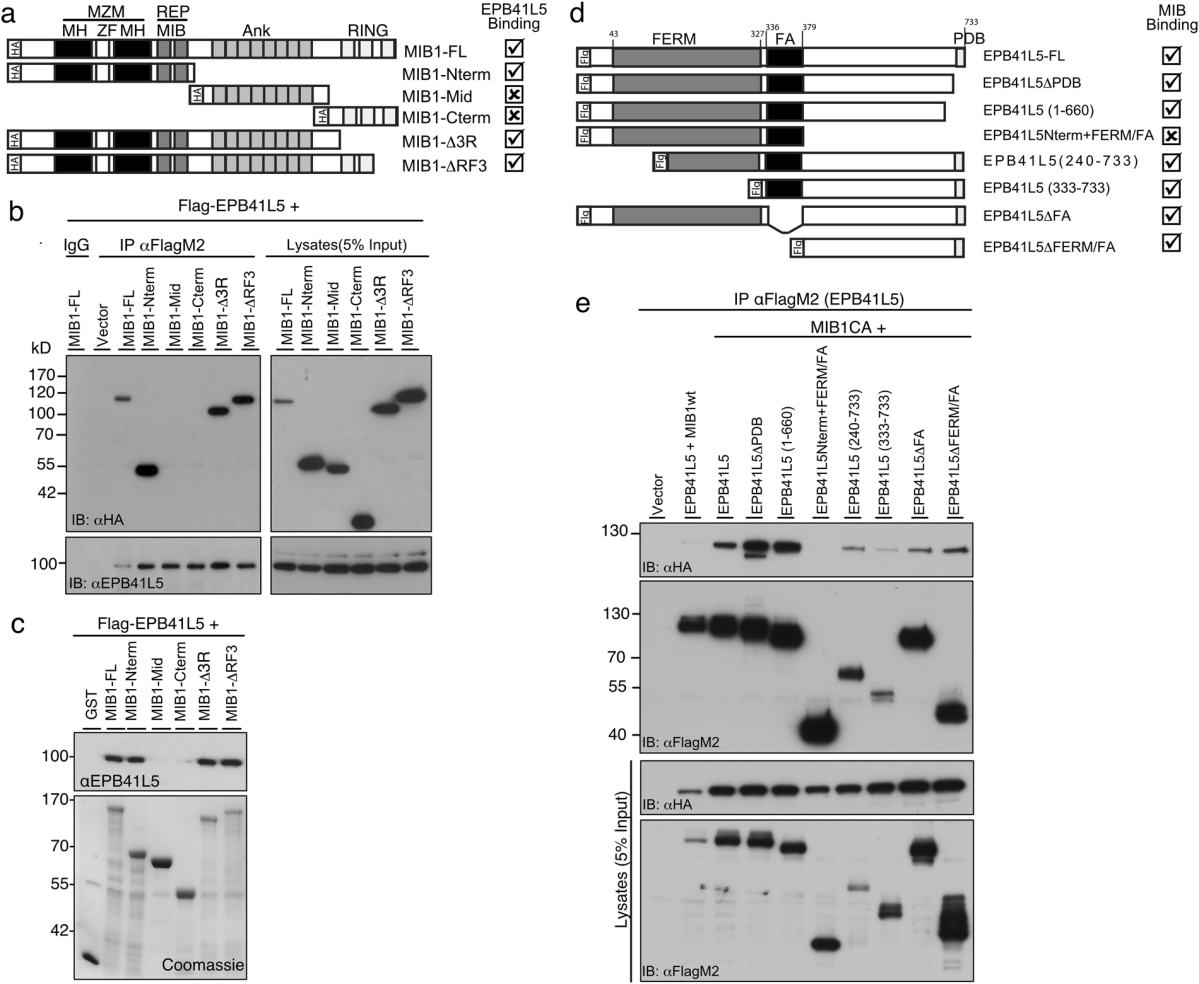
MIB1 MZM/REP region is required for binding to EPB41L5. (**a**) Schematic of the MIB full length (FL) and truncation mutant proteins displaying various domains as follows: MZM [MIB/HERC2 domains (MH); Zinc finger (ZF)]; REP [MIB domain (MIB)]; Ankyrin repeats (Ank); RING fingers; HA, Hemagglutinin tag. MIB1-FL and truncated mutants were used to map the interaction site of MIB1 with EPB41L5 by co-immunoprecipitation experiments. (**b**) The N-terminus of MIB1 mediates association with EPB41L5. Co-immunoprecipitation experiments were performed on lysates from HEK293T cells co-transfected with FLAG-EPB41L5 and the indicated HA-tagged MIB1 mutant. EPB41L5 co-immunoprecipitates MIB1-FL, MIB1-Nterm, MIB1-∆3R and MIB1-∆RF3. The panel on the right shows the relative expression of EPB41L5 and MIB1 constructs respectively, in whole cell lysates. (**c**) EPB41L5 binds to FL and N-terminus of recombinant GST-tagged MIB1. GST pulldown experiments were performed using GST-tagged MIB1-FL and mutants as described in (**a**), with lysates prepared from HEK293T cells transiently transfected with Flag-EPB41L5. EPB41L5 bound to GST-fusion proteins was identified by immunoblotting with anti-EPB41L5. The amount of GST-fusion protein used is shown in the coomassie-stained blot. (**d**) Schematic representation of the EPB41L5 full length (FL) and truncation mutant proteins displaying the FERM domain and PDZ-binding motif (PDB). (**e**) EPB41L5 co-immunoprecipitates MIB1 and the N-term + FA region of EPB41L5 is not required to mediate this interaction. Co-immunoprecipitation experiments were performed on lysates from HEK293T cells co-transfected with the FLAG-EPB41L5-FL or truncation mutants, and HA-MIB1 CA RING mutant (MIB1CA). The MIB1CA mutant binds EPB41L5, and was used in these experiments because, unlike MIB1WT, it is not degraded. Anti-FlagM2 immunoprecipitates were immunoblotted for bound MIB1 using anti-HA (top panel). All EPB41L5 deletion mutants except that lacking the C-terminus (EPB41L5Nterm + FERM/FA) bound MIB1CA. The lower panels show the relative expression of Flag-EPB41L5 and HA-MIB1 constructs, in whole cell lysates. For clarity, some blots were cropped. The full length blots are shown in Supplementary Fig. S4.

Binding of MIB1 to EPB41L5 was confirmed by co-transfection co-immunoprecipitation experiments and a series of MIB1 truncation mutants was used to map the region required for interaction with EPB41L5 (Fig. 6a,b). The amino terminal substrate binding domains of MIB1 including the MZM and REP regions was sufficient to immunoprecipitate EPB41L5 (Fig. 6b). We further confirmed these results using recombinant GST-tagged MIB1 proteins to pulldown Flag-EPB41L5 transiently expressed in HEK293T cells (Fig. 6c). In reciprocal co-immunoprecipitation experiments, MIB1 co-immunoprecipitated with full length EPB41L5 as well as EPB41L5 truncation mutants lacking the carboxy-terminal PDZ domain binding motif or the N-terminus of EPB41L5 encompassing the FERM domain and FA region (Fig. 6d,e) indicating that a region of EPB41L5 between amino acids 379 and 660 is required for binding to MIB1.

### MIB1 binds and ubiquitinates the apical polarity protein CRB1

In order to further validate MIB1 as an EPB41L5 interacting protein we generated HEK293T cells lines stably expressing FLAG epitope tagged EPB41L5 (FLAG-EPB41L5). Immuno-affinity purification was used to isolate FLAG-EPB41L5 associated proteins, and these were subjected to trypsin digestion and proteins identified by mass spectrometry. We identified unique peptides corresponding to MIB1 as well as several EPB41L5-associated proteins and proteins with known functions in cell polarity or junction formation including CRB1, the beta subunit of the Na/K ATPase, and the tight junction proteins ZO1 and ZO2 (Supplementary Fig. S2).

**Figure 7:**
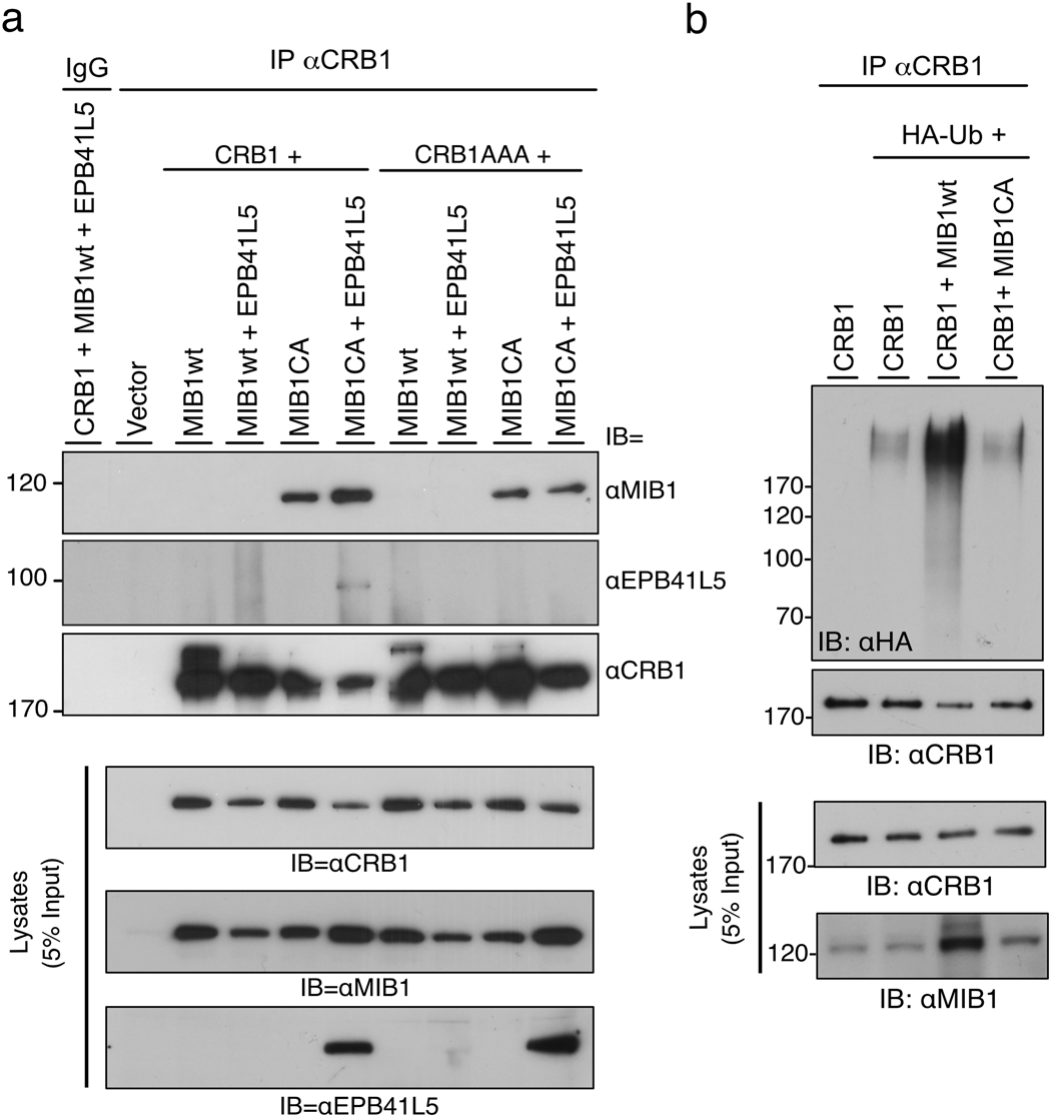
Apical polarity protein CRB1 is associated with and ubiquitinated by MIB1. (**a**) CRB1 co-immunoprecipitates with MIB1. Co-immunoprecipitation experiments were performed on lysates from HEK293T cells co-transfected with WT or mutant constructs of CRB1 and MIB1. CRB1AAA represents a mutant construct where the FERM domain binding motif of CRB1 is mutated thereby abolishing its binding to EPB41L5. The panels below (Lysates) show the relative expression of CRB1, MIB1 and EPB41L5 constructs in cell lysates. The MIB1CA ligase dead mutant bound to CRB1 and binding was enhanced in the presence of EPB41L5. (**b**) CRB1 is a substrate for MIB1-mediated ubiquitination. HEK293T cells were transiently co-transfected with myc-CRB1, HA-ubiquitin and either T7-MIB1WT or the ligase mutant T7-MIB1CA. Equivalent amounts of protein lysates were immunoprecipitated with anti-CRB1 and immunoblotted with anti-HA to detect ubiquitinated proteins. Lysates (5% of that used for immunoprecipitation) were immunoblotted with anti-CRB1 and anti-MIB1 to confirm protein expression. For clarity, blots were cropped. The full length blots are shown in Supplementary Fig. S4.

Previous studies have shown that EPB41L5 functions as part of an epithelial polarity complex that promotes lateral membrane formation and negatively regulates the activity of the apical membrane determinant, CRB1^37,38^. Therefore, we tested whether CRB1 might also be a MIB1 target in a complex with EPB41L5. In co-immunoprecipitation experiments, wild type MIB1 did not form a stable complex with CRB1 in the presence or absence of over expressed EPB41L5 (Fig. 7a). In contrast, the MIB1CA mutant, which lacks E3 ligase activity, bound to CRB1 and binding was enhanced in the presence of co-precipitating EPB41L5. Mutation of the EPB41L5 FERM domain-binding site in CRB1 (CRB1AAA) diminished, but did not abolish MIB1CA binding to CRB1 (Fig. 7a). In addition, MIB1 binding to CRB1AAA was not enhanced by over expression of EPB41L5 suggesting that MIB1 binds to CRB1 independently of EPB41L5 but that the interaction is enhanced in the presence of EPB41L5. Wild type MIB1 did not co-immunoprecipitate with CRB1 under the conditions tested either as a consequence of MIB1-mediated EPB41L5 ubiquitination and degradation, or, alternatively, autoubiquitination and degradation of HA-MIB1. Co-transfection of MIB1, CRB1 and HA-ubiquitin revealed that CRB1 is also ubiquitinated by MIB1WT  (Fig. 7b). However, in contrast to EPB41L5, ubiquitination did not appear to result in the degradation of CRB1 (Fig. 7b).

### MIB1 localizes with EPB41L5 at the basolateral membrane and ZO1 at tight junctions in polarized epithelial cells

**Figure 8:**
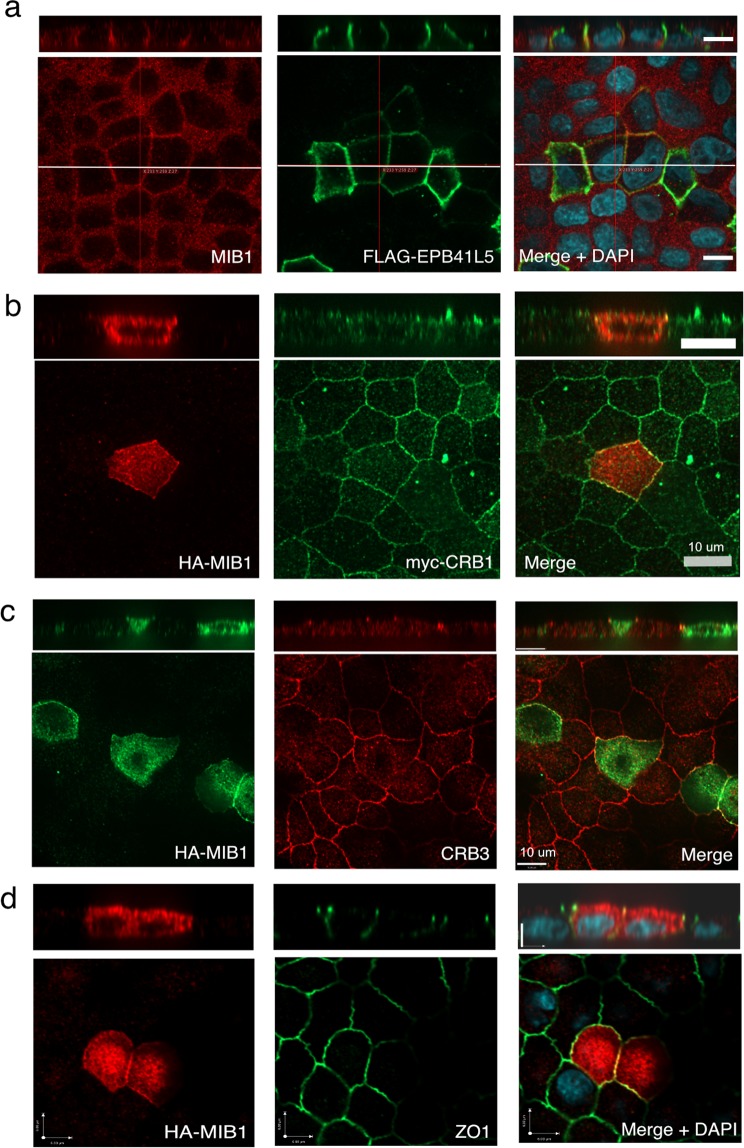
MIB1 co-localizes with EPB41L5 at the lateral membrane and CRB1, CRB3 and ZO1 at tight junctions in polarized MDCK cells. (**a**) Endogenous MIB1 is relocalized from the cytosol/tight junction to the lateral membrane by overexpression of EPB41L5. Polarized MDCK cells were transiently transfected with Flag-EPB41L5 and fixed and stained for endogenous MIB1 (left panel) and Flag (EPB41L5; middle panel). Shown are XY sections captured midway between the basal and apical membranes. XZ sections were captured where the crosshair is positioned (indicated by white line). Images were acquired at 60X magnification on a Quorum spinning disk microscope. Scale bars indicate 10 μm. (**b**) MIB1 colocalizes with CRB1 at the tight junction region of polarized MDCK cells. MDCK-MycCRB1 cells, transiently transfected with HA-MIB1, were fixed and immunostained with anti-HA (MIB1, left image) and anti-myc (myc-CRB1, middle image). Shown are XY sections taken across the apical region of the cell monolayer and corresponding XZ section. Scale bars indicate 10 μm. (**c**) MIB1 colocalizes with endogenous CRB3. Polarized MDCK cells, transiently transfected with HA-MIB1, were fixed and immunostained with anti-HA (MIB1) and anti-CRB3. Shown are XY sections taken at the apical region of the cell monolayer with a corresponding XZ section. Scale bars indicate 10 μm. (**d**) MIB1 colocalizes with endogenous ZO1 at the tight junction. Polarized MDCK cells, transiently transfected with HA-MIB1, were fixed and immunostained with anti-HA (MIB1) and anti-ZO1. Shown are XY sections taken at the apical region of the cell monolayer and corresponding XZ section. Scale bars indicate 6 μm.

In order to investigate the interaction of EPB41L5 and MIB1 in the context of polarized cells, MDCK II cells grown on permeable filter supports were transfected with EPB41L5, allowed to polarize and analysed for MIB1 subcellular distribution by immunofluorescence microscopy. In polarized cells, both endogenous and exogenously expressed EPB41L5 localizes to the basolateral membrane (Fig. 8a)^36,37^. The majority of endogenous MIB1 was cytosolic and localized to diffuse cytosolic vesicles, while in cells over expressing EPB41L5, cytosolic MIB1 was substantially decreased and lateral membrane staining was increased, suggesting MIB1 redistribution induced by EPB41L5 over expression (Fig. 8a). Similarly, in MDCK cells co-transfected with MIB1 or MIB1 truncation mutants, over-expressed MIB1 showed a diffuse cytosolic distribution (Supplementary Fig. S3, left panel), whereas when co-expressed with EPB41L5, MIB1 displayed lateral membrane localization and co-localized with EPB41L5. The MIB1 amino terminal region that binds to EPB41L5 was sufficient for localization to the lateral membrane while MIB1(C-ter) and MIB1(Mid) truncations remained cytosolic (Supplementary Fig. S3). Taken together, these results indicate that EPB41L5 is able to regulate MIB1 redistribution to the lateral membrane compartment in polarized cells and that this requires the MIB1 binding region of EPB41L5. During the course of this study, similar co-localization data were reported by Matsuda *et al*.^39^ and our findings extend these observations by showing that relocalization of MIB1 by EPB41L5 occurs in a manner that does not depend on MIB1 E3 ligase activity.

Although overexpressed MIB1 in MDCK cells was primarily cytosolic, we also observed MIB1 staining at the apical tight junction region of the lateral membrane, forming a thin band around the apical membrane. Since CRB family proteins are also localized to the apical membrane and at the tight junction we examined whether MIB1 also colocalized with Myc-tagged CRB1 expressed in MDCK cells. Partial overlap of MIB1 and Myc-CRB1 staining was observed in the apical region of cell-cell junctions consistent with localization to the tight junction (Fig. 8b). In addition, co-localization of MIB1 with the related CRB3 protein that is endogenously expressed in MDCK cells was also observed (Fig. 8c). The tight junction protein ZO1 was identified as a proximity interactor of MIB1, suggesting that these proteins may also co-localize. Staining of endogenous ZO1 in HA-MIB1 expressing MDCK cells, revealed clear co-localization of the two proteins at the tight junction region (Fig. 8d).

### MIB1 regulates polarized MDCK cell morphology

**Figure 9:**
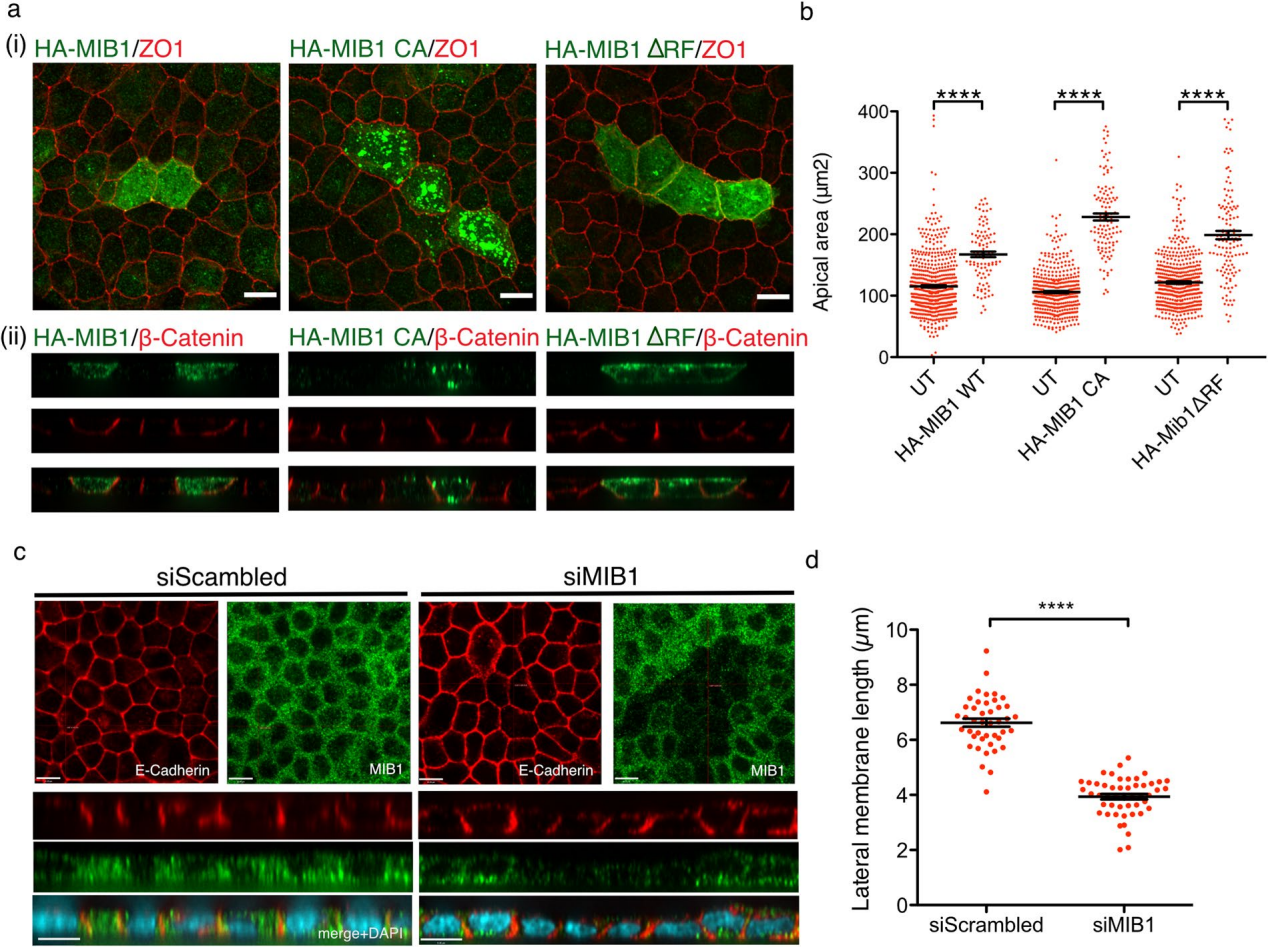
MIB1 expression in polarized MDCK cells regulates cell shape. (**a**,**b**) Overexpression of MIB1 results in the expansion of the apical membrane. MDCK cells were transfected with HA-MIB1, or the ubiquitin ligase mutants HA-MIB1CA or HA-MIB1ΔRF. Polarized cells were fixed and stained with anti-HA (MIB1-transfected cells) and either (i)anti- ZO1 to delineate the apical region or (ii) β-Catenin to show the lateral membrane. (i) XY extended focus projections of transfected cells co-stained for MIB1 and ZO1. (ii) XZ optical sections of transfected cells co-stained for MIB1 and β-catenin. Images were acquired at 60X magnification on a Quorum spinning disk microscope. Scale bar indicates 10 μm. (**b**) Quantification of the apical areas of polarized MDCK cells over expressing HA-MIB1 and adjacent cells. ZO1 staining was used to define the apical region and the area was measured using Volocity software. The individual cell measurements for 3 independent experiments are shown (HA-MIB1: 101 cells, adjacent untransfected: 601 cells; HA-MIB1CA: 112 cells, adjacent untransfected: 413 cells; HA-MIBΔRF1: 127 cells, adjacent untransfected: 477 cells) together with the corresponding mean value +/−SEM. Differences in area between transfected cells and corresponding adjacent cells were tested for statistical significance using a 2-tailed Mann Whitney test, p = < 0.0001. (**c**,**d**) Depletion of MIB1 causes the shortening of the lateral membrane in MDCK cells. (**c**) MDCK cells treated with either scrambled or MIB1 siRNAs were allowed to polarize, then fixed and stained with anti-E-Cadherin (a marker of the lateral membrane) and anti-MIB1, and counterstained with DAPI to visualize nuclei. Shown are XY optical sections taken across the middle of the cell monolayer and XZ sections taken where the crosshair is positioned (top and bottom of each panel, respectively). Images were acquired at 60X magnification on a Quorum spinning disk microscope. Scale bars indicate 10 µm. (**d**) Quantification of the effect of MIB1 depletion on lateral membrane height. Measurement of the lateral membrane length was performed using E-Cadherin stained samples and Volocity measurement software. The individual cell measurements for 2 independent experiments are shown (siScrambled: 43 cells, siMIB1: 49 cells) with the corresponding mean value +/- SEM. Statistical significance was tested using the 2-tailed Mann-Whitney test, p = <0.0001.

To test whether MIB1 expression alters polarized cell morphology, MDCK cells grown on permeable filter supports were transiently transfected with HA-MIB1, HA-MIB1CA or HA-MIB1∆RF. Cells were allowed to polarize then fixed and stained for HA, ZO-1 (tight-junctions marker) and ß-Catenin (lateral membrane marker). Cell morphology was examined by immunofluorescence confocal microscopy (Fig. 9a). Analysis of the images revealed enlarged apical areas in cells over expressing MIB1, but also in cells over expressing MIB1CA, and MIB1∆RF. Using ZO-1 to define apical surface borders, we measured apical cell areas of over expressing MIB1 or MIB1 mutants and compared them to apical areas of adjacent, non-over expressing cells. We found that polarized MDCK cells over expressing MIB1 have a significantly enlarged apical area compared to neighbouring, non over expressing cells and that expression of MIB1 mutants lacking ubiquitin ligase activity had a similar effect (Fig. 9b). These data indicate that over expression of MIB1 in polarized MDCK cells induces apical area enlargement and that these changes may be through both ligase dependent and independent mechanisms.

In order to further study the role of MIB1 in cell morphology and cell polarity, we tested the effects of transiently depleting MIB1 in polarized cells. MDCK cells transfected with scrambled or MIB1 siRNAs, were allowed to polarize and were then stained for E-Cadherin (lateral membrane marker) and endogenous MIB1, and analysed by confocal microscopy (Fig. 9c). The XZ images show that cells transiently depleted of MIB1 display reduced lateral membrane height and appeared flatter than neighbouring cells not depleted of MIB1 or of cells treated with scramble siRNA (Fig. 9c, bottom). In order to quantify the observed lateral membrane reduction, the lateral membrane lengths of cells treated with scrambled or MIB1 siRNAs were measured using E-Cadherin stained samples and Volocity analysis software. Data analysis revealed a statistically significant reduction in the lateral membrane length of polarized MDCK cells depleted of MIB1. Taken together, these data indicate that MIB1 is important for lateral membrane maintenance in polarized MDCK cells.

## Discussion

Here we used proximity dependent biotinylation (BioID) to define the MIB1 interactome and confirm several proximity interactors as putative substrates of MIB1 E3 ligase activity. The BioID approach can overcome several limitations associated with the identification of ubiquitin ligase interaction partners and putative substrates by identifying closely associated proteins including weak and transient interactions. In addition, the list of proteins identified also provides a map of MIB1 cellular localization and possible involvement within known functional complexes.

The MIB1 BioID screen identified 163 proteins. Of these, 27 have been identified as MIB1 interactors in other studies via by AP-MS, AP-Western or 2-Hybrid screens^18,40,41^, 6 of which have been confirmed as a direct interaction^18,40^ or ligase substrates: PCM1, AZI1^14,15^, CTNND1^17^, TBK1^42^, and USP9x^43^. Thus, the majority of proteins identified here are unique and expand the network of known MIB1-interactors, highlighting potential new functions and substrates. Our data further confirm the localization of MIB1 at centrioles/satellites through identification of a large network of centriolar/centriolar satellite proteins. Similarly, the proximity interactions identified provide supporting evidence for MIB1 function in endocytosis and vesicular trafficking, and suggests roles in RNA and DNA processing, and cell-cell adhesion.

Several proteomic mass spectroscopy analyses identified MIB1 as a component of the centrosome/centrosomal satellite, either as a component of purified centrosomal preparations or via affinity purification with a known centrosomal protein^11–14,44^. Our screen confirmed previously identified, MIB1-associated, centrosome/centrosomal satellite proteins, including PCM1, AZI1 and OFD1. Both PCM1 and AZI1 have been shown to be substrates of MIB1 activity^14,15^, though stability of these proteins in response to ubiquitination differed between studies. Although not identified in our study, PLK4 and TALPID3, proteins involved in centriolar duplication and ciliogenesis respectively, have also been reported to be ubiquitinated by MIB1^15,16^. We show here that CCDC14 and KIAA0753, proteins implicated in centriole duplication^11,45^, are also likely MIB1 substrates. Under basal conditions with no proteosomal inhibition, CCDC14 exhibited a clear MIB1-mediated increase in molecular weight with no apparent degradation. KIAA0753 was similarly resistant to degradation. Both proteins exhibited incorporation of HA-ubiquitin. CCDC14 and KIAA0753 influence centriole duplication antagonistically through opposite effects on CEP63 localization to the centriolar satellite. It remains to be determined whether ubiquitination of CCDC14 and/or KIAA0753 by MIB1 also regulates centriolar duplication.

In vertebrates, MIB1 plays an essential role in the activation of Notch signalling through the ubiquitination and endocytosis of Notch ligands, which triggers the proteolytic events releasing the Notch intracellular domain^2,6,7^. In keeping with the well-established role of MIB1 in Notch ligand endocytosis we identified several MIB1 interactions with vesicle trafficking proteins, considerably enhancing the list of proteins previously identified by either AP-MS or Yeast 2-Hybrid screening methods^18,40,41^. We further expand this list to include ESCRT-0 proteins involved in traffic of ubiquitinated membrane proteins to multivesicular bodies for subsequent degradation within the lysosome (HGS, STAM, STAM2)^46^, and proteins involved in endosome-lysosome fusion and autophagy (TBC1D5, TBC1D15, AGFG1, SQSTM1/p62)^47–49^. The Eps15/FCHO2/AP2B1 complex functions early in the formation of clathrin coated vesicles^34,35^ and we find that EPS15 and FCHO2 are targets for ubiquitination by MIB1. Eps15 is also targeted for mono-ubiquitination by Nedd4^50^ and Parkin^51^, which bind Eps15 via its ubiquitin interaction motif. It will be interesting to determine if MIB1, which is auto-ubiquitinated, binds in a similar manner to Eps15, and whether Eps15 ubiquitination by MIB1 mediates endocytosis of Notch ligands, or additional transmembrane proteins identified in this study: EphA2, ANTRX1, LPHN2 and TMEM57. Indeed, mono-ubiquitinated Eps15 is required for EGFR internalization and is a target of the deubiquitinase USP9x^33^, also identified in this study as a proximity interactor of MIB1, and by others^18,40,41,43^.

Several of the MIB1 proximity interactors tested as potential MIB1 substrates paradoxically exhibited an increase in abundance in cell lysates when co-expressed with MIB1, in particular AP2B1, OFD1 and ZO1. It is possible that MIB1 activity may increase the stability of these protein indirectly via the ubiquitination and subsequent degradation of other E3 ligases which themselves normally ubiquitinate these proteins causing degradation. Indeed, a number of proteins involved in mediating ubiquitin- and ubiquitin-related processes were identified in our screen.

Our studies also identified the polarity protein EPB41L5 as a MIB1 substrate and suggest that MIB1 could function as a regulator of cell morphology in part through ubiquitination and degradation of EPB41L5. Furthermore, MIB1 localization to the basolateral membrane is regulated by EPB41L5 in the context of polarized epithelial cells.

When expressed in HEK293T or HeLa cells, ubiquitination by MIB1 leads to degradation of EPB41L5 and reversal of the phenotypic effects of EPB41L5 overexpression. However, while MIB1 appears to degrade exogenous EPB41L5 in MDCK cells we are unable to conclude that degradation of EPB41L5 is required for the observed effects of MIB1 expression on polarized cell morphology. Co-expression results in MIB1 relocalization from the cytosol vesicles to the basolateral membrane where it co-localizes with EPB41L5. However, co-expression of WT MIB1 and EPB41L5 was only observed in rare cells, though in all cases they colocalized. This observation suggests that at the lateral membrane EPB41L5 is not targeted for degradation by MIB1. While polarized activation has not been reported, MIB1 activity can be regulated by PAR-1 mediated phosphorylation^52^. The PAR-1 serine/threonine kinase acts as a polarity protein down stream of atypical-PKC and is required for establishing apical- basal membrane domains^53^. Phosphorylation of MIB1 by PAR1 leads to its degradation, presumably by auto-ubiquitination, and as a consequence reduces Notch signalling^52^. Whether PAR-1 regulates asymmetric or polarized distribution of activated MIB1 is unknown. Alternatively, EPB41L5 may be stabilized at the lateral membrane via the activity of a deubiquitinase. One candidate is USP9x, which forms a complex with MIB1, increasing its stability by deubiquitination^43^. That USP9x may similarly stabilize EPB41L5 at the lateral membrane is supported by reports that it deubiquitinates other adhesion junction proteins, including EFA6, AF-6, β-catenin and E-Cadherin^54–57^.

CRB is a transmembrane protein that is required for apical membrane formation in polarized cells^58–64^. While EPB41L5 functions as a negative regulator of the CRB apical polarity complex, the mechanisms by which it regulates CRB1 activity are not well understood. Loss of EPB41L5 orthologue function in zebrafish (Mosaic eyes) or *Drosophila* (YURT), leads to changes in CRB levels and subcellular distribution, while in the mouse *limulus* mutant, CRB localization appears unaffected early in development^36,37,65^. We show that CRB1 binds to, and is a substrate of, MIB1, and that in MDCK cells exogenous MIB1 colocalizes with CRB1 and CRB3. Overexpression of MIB1 in MDCK cells causes expansion of the apical membrane, suggesting that MIB1 may regulate apical membrane size by influencing CRB activity. In Drosophila, the E3 ligase neuralized has been demonstrated to regulate Crumbs endocytosis and trafficking through ubiquitin-mediated degradation of stardust, a member of the MAGUK family of adaptors that binds to Crumbs^66,67^. Since EPB41L5 binds to CRB, and is a negative regulator of its activity^37^, we speculate that MIB1 could similarly mediate its effect through its ligase-dependent degradation of CRB-associated EPB41L5. Alternatively, MIB1 may affect CRB activity directly by ubiquitination, analogous to its role in regulating Notch ligand Delta ubiquitination and endocytosis^2,68,69^. Endosomal trafficking is also a mechanism to regulate the amount and localization of CRB^66,70–72^. Since MIB1 forms many connections with the endocytic machinery, the effects of MIB1 on apical expansion may also be a consequence of ubiquitin dependent alterations in trafficking of CRB or another apical membrane protein. Finally, as both EPB41L5 and CRB1 are ubiquitinated by MIB1, it will be important to determine if they act competitively as substrates since recent studies in zebrafish have suggested that EPB41L5 competition with Delta for MIB1 binding prevents MIB1-mediated ubiquitination of EPB41L5 causing its stabilization^39^. Finally, our data show it is likely that MIB1 has E3 ligase independent effects on epithelial cell morphology, potentially by acting as a scaffold or adaptor protein.

In conclusion, we report a comprehensive interactome for the E3 ubiquitin ligase MIB1 highlighting additional interactions related to its annotated functions in centrosome and cilia as well as endocytosis and vesicle trafficking, and suggesting a potential role in DNA and RNA processing. Our findings also reveal a novel role for MIB1 as a regulator of epithelial polarity and morphology, and association with polarity complex proteins.

## Materials and Methods

### BioID

FlagBirA-MIB1 was constructed by PCR cloning of full length human MIB1 into the pcDNA5 FRT/TO Flag BirA* vector. A stable inducible cell line was made by cotransfection of FlagBirA-MIB1 with pOG44 Flp recombinase into Flp-In 293 T-REx host cells, using Lipofectamine 2000 transfection reagent (Invitrogen). Stable cells were selected for in 200 μg/ml hygromycin and pooled. A control cell line was made by transfection with pcDNA5 FRT/TO FlagBirA* vector.

Preparation of cells for Biotin-Streptavidin affinity purification of biotin-labelled proteins: Flag-BirA-MIB1 and Flag-BirA Flp-In 293 T-REx cells were each grown to approximately 60% confluency in tetracycline-free media in 5 × 150 mm dishes. 24 hours before harvest, FlagBirA-MIB1 expression was induced by addition of 1 μg/ml doxocycline (Sigma-Aldrich D989) in the presence of 50 μM biotin (Sigma-Aldrich B4639). Cells were scraped into cold PBS, combined and washed twice with PBS at 4 °C. Cell pellets were flash frozen and stored at −80 °C.

#### Biotin-streptavidin affinity purification

The frozen cell pellet was resuspended in 10 mL of lysis buffer (50 mM Tris-HCl pH 7.5, 150 mM NaCl, 1 mM EDTA, 1 mM EGTA, 1% Triton X-100, 0.1% SDS, 1:500 protease inhibitor cocktail (Sigma-Aldrich), 1:1000 benzonase nuclease (Novagen)), incubated on an end-over-end rotator at 4 °C for 1 hr, briefly sonicated to disrupt any visible aggregates, then centrifuged at 45,000 × *g* for 30 min at 4 °C. The supernatant was transferred to a fresh 15 mL conical tube, 30 uL of packed, pre-equilibrated streptavidin-sepharose beads (GE) were added, and the mixture incubated for 3 hr at 4 °C with end-over-end rotation. Beads were pelleted by centrifugation at 2000rpm for 2 min and transferred with 1 mL of lysis buffer to a fresh Eppendorf tube. Beads were washed once with 1 mL lysis buffer and twice with 1 mL of 50 mM ammonium bicarbonate (pH 8.3). Beads were transferred in ammonium bicarbonate to a fresh centrifuge tube, and washed two more times with 1 mL ammonium bicarbonate buffer. Tryptic digestion was performed by incubating the beads with 1 μg MS grade TPCK trypsin (Promega, Madison, WI) dissolved in 200 uL of 50 mM ammonium bicarbonate (pH 8.3) overnight at 37 °C. The following morning, an additional 0.5 μg trypsin was added, and the beads incubated 2 hr at 37 °C. Beads were pelleted by centrifugation at 2000 × *g* for 2 min, and the supernatant was transferred to a fresh Eppendorf tube. Beads were washed twice with 150 uL of 50 mM ammonium bicarbonate, and these washes were pooled with the first eluate. The sample was lyophilized, and resuspended in buffer A (0.1% formic acid). 1/5th of the sample was analyzed per MS run.

#### Mass spectrometry analysis

Analytical columns (75-um inner diameter) and pre-columns (150-um inner diameter) were made in-house from fused silica capillary tubing from InnovaQuartz (Phoenix, AZ) and packed with 100 Å C18–coated silica particles (Magic, Michrom Bioresources, Auburn, CA). Peptides were subjected to liquid chromatography (LC)-electrospray ionization-tandem mass spectrometry, using a 120 min reversed-phase (100% water–100% acetonitrile, 0.1% formic acid) buffer gradient running at 250 nl/min on a Proxeon EASY-nLC pump in-line with a hybrid LTQ-Orbitrap Velos mass spectrometer (Thermo Fisher Scientific, Waltham, MA). A parent ion scan was performed in the Orbitrap using a resolving power of 60,000, then up to the twenty most intense peaks were selected for MS/MS (minimum ion count of 1000 for activation), using standard collision induced dissociation fragmentation. Fragment ions were detected in the LTQ. Dynamic exclusion was activated such that MS/MS of the same m/z (within a range of 15 ppm; exclusion list size = 500) detected twice within 15 s were excluded from analysis for 30 s. For protein identification, Thermo. RAW files were converted to the.mzXML format using Proteowizard^73^, then searched using X!Tandem^74^ against the human (Human RefSeq Version 107) database. X!Tandem search parameters were: 15ppm parent mass error; 0.4 Da fragment mass error; complete modifications, none; cysteine modifications, none; potential modifications, +16@M and W, +32@M and W, +42@N-terminus, +1@N and Q. Data were analyzed using the trans-proteomic pipeline (TPP)^75,76^ via the ProHits software suite^77^. Proteins identified with a TPP cut-off of 0.9 (corresponding to ≤1% FDR) and at least two unique peptides were analyzed with SAINT Express v.3.3. Ten control runs (from cells expressing the FlagBirA* epitope tag) were collapsed to the two highest spectral counts for each prey, and compared to the two highest spectral counts between four replicates of MIB1 BioID. High confidence interactors were defined as those with BFDR ≤ 0.01. All raw mass spectrometry files have been uploaded to the public data repository MassIVE (massive.ucsd.edu), accession # MSV000083085.

#### Networks analysis

SAINT data were uploaded to string-db.org for network analysis. The resulting network was imported into Cytoscape 3.6.1 (http://www.cytoscape.org) for figure preparation.

### Plasmid constructs & mutagenesis

Full length expression construct for human myc-tagged CRB1 (hCRB1), obtained from the laboratory of Dr. B. Margolis (University of Michigan Medical School, Ann Arbor, MI), was cloned into the pSecTag2B vector (Invitrogen) as previously described^78,79^. Mutant hCRB1 expression construct was generated using the QuikChange II site directed mutagenesis (Stratagene) and harbors point mutations (Y1398A; P1340A; E1405A) within the FERM domain binding motif. Full length human cDNA of EPB41L5 (hEPB41L5) was obtained by PCR from a cDNA template (BC054508; Open Biosystems) and then subsequently cloned into pFLAG-CMV2 (Sigma-Aldrich) with an amino-terminal FLAG epitope tag. Deletion mutant constructs of FLAG-hEPB41L5 were also cloned by PCR and include the following variations: hEPB41L5 [(aa 1-334), (aa 1-506), (aa 1-660), (aa 240-733), (aa 333-733)], hEPB41L5ΔFA (aa 1-733Δ336-379), hEPB41L5ΔFERM/FA (379-733), hEPB41L5ΔPDB (aa 1-728), hEPB41L5Nterm + FERM/FA (aa 1-378). A full length hMIB1 cDNA was obtained by PCR from two cDNA templates [AK091266; Biological Resource Center (NBRC), NITE, Japan and RZPDp9016M1220Q2; B-Bridge International Inc., USA)] and cloned into the pEF(HA)_2_ vector. A hMIB1 E3 ubiquitin ligase dead mutant construct was generated using the QuikChange II site directed mutagenesis (Stratagene), in which the cysteine residue at position 985 known to confer ligase activity was mutated to an alanine (C985A). Truncated mutant forms of hMIB1 (N-terminal domain: aa 1-410; Middle domain: aa 411-788; C-terminal domain: aa 788-1006; hMIB1ΔRF1-3: aa 1-818; hMIB1ΔRF3: aa 1-962) were cloned into pEF(HA)_2_ and pGEX-4T1 (Clontech) with an amino terminal GST epitope tag. All cDNA expression constructs and mutations generated by PCR were verified by sequencing.

BirA-tagged hMIB1 cDNA was constructed by PCR from HA-hMIB1 template and cloned into pcDNA5 FLAG/FRT/TO-hBirA. ZO1-myc in pCB6 was a gift from James Anderson and Alan Fanning (Addgene plasmid#30317^80^). For Flag-CCDC14, full length CCDC14 (BC040285) was obtained by PCR of human CCDC14/pDestcV5 cDNA (SPARC Biocentre, Sickkids) and cloned into p3XFlag CMV-10. KIAA0753-Flag was similarly constructed using a human KIAA0753 (BC113016.1) MGC clone cDNA template (SPARC Biocentre, Sickkids) which was cloned into p3XFlag-CMV-14. Eps15-Flag was constructed by cloning full length human Eps15, obtained by PCR from hEps15-pmCherryN1 which was a gift from Christien Merrifield (Addgene plasmid#27696^81^), and cloned into p3XFlag CMV-14. Human Flag-FCHO2 was constructed by PCR from FCHO2/pCR4-TOPO (Harvard PlasmID ID HsCD00342448) and cloned into p3XFlag-CMV-10. Human AP2B1 (BC006201) in pDESTcHA was obtained from SPARC Biocentre, Sickkids, Toronto.

### Cell culture, transfection and generation of HEK293T pFLAG-EPB41L5 stable lines

HEK293T and HeLa cells were cultured in Dulbecco’s Modified Eagle Medium (DMEM) (Wisent Inc.) supplemented with 10% fetal bovine serum and MDCK II cells were maintained in DMEM supplemented with 5% FBS. For plasmid transfection, Lipofectamine 2000 reagent (Invitrogen) was used according to the manufacturer’s instructions. Cells were cultured at 37 °C for 30–48 h before lysis. In order to generate stably transfected HEK293T cell lines, cells were transfected using Lipofectamine 2000 (Invitrogen) with 2 µg of FLAG-EPB41L5 cDNA or control pFLAG empty vector. After selection with 2 μg/ml of Puromycin for two weeks, clones were isolated and propagated to generate stable cell line clones.

### HeLa cell transfection and cell morphology assay

HeLa cells were onto 6 well dishes and transiently transfected with Flag-EPB41L5/pIRES/EGFP (0.6μg) plus T7/pcDNA3.1 (0.9 μg), Flag-EPB41L5/IRES/EGFP plus T7-MIB1 (0.9 μg), or T7-MIB1 (0.9 μg) with empty pIRES2/EGFP (0.6 μg) using Lipofectamine2000 (4.5 μl). After 18 hours cells were trypsinized and reseeded onto glass coverslips coated with 5 μg/ml fibronectin, and allowed to adhere for 2 hours. Cells were fixed in 2% PFA, permeablized with 0.1% TritonX-100 and stained with mouse anti-FlagM2 (EPB41L5), rabbit anti-GFP (co-expressed GFP), or guinea pig anti-MIB1 (MIB1), and AlexaFluor 635 phalloidin (F-actin). Images were captured using a 20X/0.75 objective on a Quorum Spinning Disc Confocal microscope. Cell morphology was quantified by determining the length/width ratio (width was measured at a 45 degree angle to the midpoint of the length line).

### Antibodies

A rabbit polyclonal antibody for hCRB1 was raised against a GST fusion protein corresponding to amino acids 255–407 within the extracellular domain of the protein. hMIB1 and mEPB41L5 polyclonal antibodies were raised against GST fusion proteins corresponding to amino acids 236–445 and 669–732 of the proteins respectively, and antisera affinity purified using the corresponding antigen. Additional primary antibodies used available from commercial sources include: HA12CA5 (Roche,IF 1:500, IB 1:1000), HA-Tag (C29F4; Cell Signaling Technology 3724; IP 5μl), Histidine (HIS.H8, Sigma-Aldrich 05-949, IB 1:500), FLAGM2 (Sigma-Aldrich, mouse, IF 1:500, IB 1:1000; Abnova, rabbit, IF: 1:500), myc9E10 (Clontech,; IF 1:500, IB 1:1000), T7tag (EMD Millipore 69522-3; IB 1:500) E-cadherin (BD Biosciences, mouse, IF 1:300), ZO1 (Zymed, mouse, IF 1:300; Millipore, rat, IF 1:500), β-catenin (Millipore, rabbit, IF 1:300), Actin-phalloidin (Molecular Probes, Invitrogen, IF 1:500), GFP (ThermoFisher Scientific A-11122; IF 1:500) and CRB3 (Sigma HPA013835, IF 1:50). Secondary antibodies conjugated with AlexaFlour 488 (Molecular Probes), Cy3 and Cy5 (Jackson Laboratories).

### *In vivo* protein abundance assay

Exponentially growing 293 T cells were seeded at 1 × 10^6^ cells per 6 well. After 4 hours, cells were transfected as follows: 0.5 μg T7- or HA-MIB1 WT, CA or Δ3 R mutant plus 0.5 μg query substrate cDNA were added together with 3ul Lipofectamine to 1 ml DMEM/10%FBS and incubated for 18 hours. Cells were lysed with 300ul 1XLaemmli sample buffer (without bromophenol blue or β-mercaptoethanol), boiled and protein concentration measured. Following addition of bromophenol blue and β -mercaptoethanol, 50 μg lysate was separated by SDS-PAGE, transferred to PDVF membrane, and immunoblotted with anti-FlagM2, anti-T7 or anti-HA to identify substrate and MIB1 as appropriate.

### Ubiquitination assay

HEK293T cells (4 × 10^6^) were seeded onto 10 cm dishes 1 day prior to transfection. For each transfection the following was used: HA-Ub (0.5 μg) or HIS6-Ub, T7-MibWt (1 μg) and query substrate cDNA (1 μg), plus 7.5ul Lipofectamine2000. After 18 hours, 10uM MG132 was added for an additional 4 hours. Cells were lysed with 1 ml RIPA + 1%SDS + protease inhibitors, boiled for 5 minutes, passed through a 26 G needle 10 times and centrifuged. Lysate (2 mg) was diluted 10 fold in NP40 Lysis Buffer and tagged-substrates were immunoprecipitated with either 4 μg anti-FlagM2 or 5μl rabbit anti-HA overnight at 4 °C. IPs were separated on 7.5% SDS-PAGE gel and ubiquitinated protein visualized using mouse anti-HA or anti-HIStag.

### Co-Immunoprecipitation and immunoblot

Transiently transfected HEK293T cells were lysed in 1 ml of ice-cold lysis buffer (50 mM Tris-HCl [pH 8.0], 150 mM NaCl, 1 mM EDTA, 0.5% Triton X-100, 10% glycerol, 100 mM sodium fluoride containing COMPLETE protease inhibitor tablet [Roche Molecular Biochemicals]) and cleared by centrifugation at 14,000 × *g* for 10 min at 4 °C. The protein concentrations of cell lysates were determined using the Bio-Rad Protein Assay (Bio-Rad Laboratories). For immunoprecipitations, one milligram of total cell lysate was incubated with 1 µg of antibody and 50 µl of a 20% (w/v) protein A or G-Sepharose bead slurry. The immune complexes were washed with lysis buffer and eluted and boiled in SDS-Laemmli sample buffer. Proteins were separated by SDS-PAGE, transferred onto PVDF membrane and immunoblotted with primary antibodies overnight at 4 °C. Bound antibodies were visualized using HRP-conjugated protein A (Bio-Rad Laboratories) or goat anti-mouse HRP and western blot analysis was performed using the enhanced chemiluminescence (ECL) system (Amersham Biosciences).

### MIB1 knockdown in MDCK II cells

Transient siRNA knockdown of MIB1 was carried out using two custom siRNA oligonucleotides (5′-GGAUAAAGAUGGAGAUAGAUU-3′) and (5′-GCAAAGUGUCAUAAGGAA-3′) purchased from Dharmacon. MDCK cells were transfected at 90% confluence with 200 pmol of the siRNA duplex or scrambled RNA using Lipofectamine 2000 (Invitrogen) according to the manufacturer’s instructions. Cells were fixed and stained 36–48 h post-transfection.

### MDCK immunocytochemistry

MDCK cells were grown to confluence on Falcon cell culture inserts after which they were fixed with 2% paraformaldehyde, 30 mM sucrose in PBS for 25 min at room temperature. Cells were permeabilized with 0.05% saponin and blocked in 5% normal donkey serum for 30 min at room temperature. Primary antibodies were applied overnight at 4 °C. Filters were washed with 0.05% Triton-X100 in PBS. Secondary antibodies were applied for 30 min at 37 °C after which cells were washed and mounted in Dako Cytomation fluorescent mounting medium. Confocal images were acquired using a Quorum spinning disk microscope at 60x or 63x magnification and image analyses (including quantifications) were performed using Volocity software.

### Statistics

GraphPad Prism 5 was used to prepare all graphs and carry out statistical analyses of data. Data sets were tested for normality using the D’Agnostino and Pearson omnibus normality test (alpha = 0.05) and non-parametric tests used for data that was not normally distributed. The statistical tests used for each data set are described in the corresponding figure legend.

## Supplementary information

Supplementary Table S1

Supplementary information

## Data Availability

All raw mass spectrometry files have been uploaded to the public data repository MassIVE (massive.ucsd.edu), accession # MSV000083085.
