# Supplementary material for: Proximity interactions of the ubiquitin ligase Mind bomb 1 reveal a role in regulation of epithelial polarity complex proteins
Source: Sci Rep. 2019 Aug 28;9:12471. doi: 10.1038/s41598-019-48902-x (PMC6713736; doi:10.1038/s41598-019-48902-x)

Proximity interactions of the ubiquitin ligase Mind bomb 1 reveal a role in regulation of epithelial polarity complex proteins

Dho, Sascha E.[1], Silva- Gagliardi, Nancy[1], Morgese, Fabio[1], Coyaud, Etienne[2], Lamoureux, Emily[1], Berry, Donna M.[1], Raught, Brian [2,3], and C. Jane McGlade[1,3] *

Supplementary Tables and Figures

1. Supplementary Table S2: GO Enrichment Analysis
2. Supplementary Figure S1. Expression of Flag-EPB41L5/IRES/GFP and T7-MIB1 in Hela cells used for morphology assay.
3. Supplementary Figure S2: Identification of EPB41L5 binding partners by LC MS/MS.
4. Supplementary Figure S3: MIB1 relocalization to the plasma membrane when co-expressed with EPB41L5 requires the EPB41L5 interaction region.
5. Supplementary Figure S4: Original western blots showing cropped regions used to prepare figures in the main text.

Supplementary Table S2: GO Enrichment Analysis

Annotation Cluster 1 — Enrichment Score: 7.757390046319508

Category	Term		Count	%	PValue	Genes	List Total	Pop Hits	Pop Total	Fold Enrichm	Bonferroni	Benjamini	FDR
GOTERM_BP_DIRECT	GO:0000086	G2/M transition of mitotic cell cycle	15	9.62	7.27982E-12	93323, 510	140	137	16792	13.13	5.47E-09	2.74E-09	1.11E-08
GOTERM_BP_DIRECT	GO:0051297	centrosome organization	9	5.77	2.09838E-10	93323, 117	140	33	16792	32.71	1.58E-07	5.26E-08	3.19E-07
GOTERM_CC_DIRECT	GO:0070652	HAUS complex	6	3.85	1.75168E-09	93323, 794	148	8	18224	92.35	4.10E-07	1.02E-07	2.25E-06
GOTERM_BP_DIRECT	GO:0051225	spindle assembly	7	4.49	7.40592E-08	93323, 794	140	27	16792	31.10	5.57E-05	1.39E-05	1.13E-04
GOTERM_BP_DIRECT	GO:0007067	mitotic nuclear division	14	8.97	1.45417E-07	93323, 794	140	248	16792	6.77	1.09E-04	2.19E-05	2.21E-04
GOTERM_BP_DIRECT	GO:0051301	cell division	16	10.26	2.12656E-07	93323, 784	140	350	16792	5.48	1.60E-04	2.67E-05	3.24E-04
GOTERM_CC_DIRECT	GO:0005874	microtubule	15	9.62	2.30558E-07	93323, 784	148	311	18224	5.94	5.39E-05	8.99E-06	2.96E-04
GOTERM_CC_DIRECT	GO:0005819	spindle	9	5.77	6.17682E-06	79848, 794	148	121	18224	9.16	0.00144434	1.81E-04	0.00794045

Annotation Cluster 2 — Enrichment Score: 4.376357251331086

Category	Term		Count	%	PValue	Genes	List Total	Pop Hits	Pop Total	Fold Enrichm	Bonferroni	Benjamini	FDR
GOTERM_BP_DIRECT	GO:0042059	negative regulation of epidermal growth factor rece	11	7.05	2.30333E-13	161, 163, 5	140	36	16792	36.65	1.73E-10	1.73E-10	3.51E-10
GOTERM_CC_DIRECT	GO:0030122	AP-2 adaptor complex	7	4.49	4.07201E-10	161, 163, 9	148	13	18224	66.30	9.53E-08	3.18E-08	5.23E-07
GOTERM_BP_DIRECT	GO:0072583	clathrin-mediated endocytosis	6	3.85	5.07274E-07	161, 163, 1	140	20	16792	35.98	3.81E-04	5.45E-05	7.72E-04
GOTERM_MF_DIRECT	GO:0035615	clathrin adaptor activity	5	3.21	8.15198E-07	161, 163, 1	137	10	16881	61.61	1.73E-04	8.64E-05	0.00103151
GOTERM_CC_DIRECT	GO:0036020	endolysosome membrane	5	3.21	1.91297E-06	161, 163, 1	148	12	18224	51.31	4.48E-04	6.39E-05	0.00245922
GOTERM_BP_DIRECT	GO:0006886	intracellular protein transport	11	7.05	2.71801E-05	161, 1020,	140	236	16792	5.59	0.02023223	0.00204188	0.0413743
GOTERM_BP_DIRECT	GO:0048013	ephrin receptor signaling pathway	7	4.49	7.88401E-05	161, 163, 6	140	86	16792	9.76	0.05756666	0.00492865	0.11996853
GOTERM_BP_DIRECT	GO:0050690	regulation of defense response to virus by virus	5	3.21	7.88908E-05	161, 163, 1	140	28	16792	21.42	0.05760258	0.00455331	0.12004562
GOTERM_BP_DIRECT	GO:0048268	clathrin coat assembly	4	2.56	0.000149392	163, 11554	140	13	16792	36.91	0.10626989	0.00699737	0.22721172
GOTERM_CC_DIRECT	GO:0030666	endocytic vesicle membrane	6	3.85	0.000191899	161, 163, 1	148	66	18224	11.19	0.04391524	0.00407429	0.24641938
GOTERM_CC_DIRECT	GO:0030669	clathrin-coated endocytic vesicle membrane	5	3.21	0.000326321	161, 163, 1	148	41	18224	15.02	0.07352803	0.00448236	0.4186976
GOTERM_BP_DIRECT	GO:0007018	microtubule-based movement	6	3.85	0.000559468	161, 163, 1	140	81	16792	8.88	0.34350333	0.02445126	0.84842145
GOTERM_BP_DIRECT	GO:0060071	Wnt signaling pathway, planar cell polarity pathway	6	3.85	0.000998721	161, 163, 1	140	92	16792	7.82	0.5283006	0.03687365	1.50982109
GOTERM_BP_DIRECT	GO:0019886	antigen processing and presentation of exogenous	5	3.21	0.007161458	161, 163, 1	140	92	16792	6.52	0.99550501	0.15540645	10.3652509
GOTERM_CC_DIRECT	GO:0030131	clathrin adaptor complex	3	1.92	0.007200785	161, 163, 1	148	16	18224	23.09	0.81567958	0.07398723	8.8720897
GOTERM_CC_DIRECT	GO:0030117	membrane coat	3	1.92	0.018503454	161, 163, 1	148	26	18224	14.21	0.98735341	0.14944437	21.3453315
GOTERM_MF_DIRECT	GO:0008565	protein transporter activity	4	2.56	0.020368871	161, 163, 1	137	72	16881	6.85	0.98725716	0.32740834	22.9255495
GOTERM_BP_DIRECT	GO:0016192	vesicle-mediated transport	4	2.56	0.131917287	161, 163, 1	140	152	16792	3.16	1	0.81522884	88.3967684

Annotation Cluster 3 — Enrichment Score: 2.9147289108024097

Category	Term		Count	%	PValue	Genes	List Total	Pop Hits	Pop Total	Fold Enrichm	Bonferroni	Benjamini	FDR
GOTERM_CC_DIRECT	GO:0016282	eukaryotic 43S preinitiation complex	4	2.56	0.000217906	8662, 8661	148	15	18224	32.84	0.04971708	0.00391505	0.27977174
GOTERM_CC_DIRECT	GO:0033290	eukaryotic 48S preinitiation complex	4	2.56	0.000217906	8662, 8661	148	15	18224	32.84	0.04971708	0.00391505	0.27977174
GOTERM_CC_DIRECT	GO:0005852	eukaryotic translation initiation factor 3 complex	4	2.56	0.00032183	8662, 8661	148	17	18224	28.97	0.07255356	0.00469645	0.41294612
GOTERM_BP_DIRECT	GO:0001731	formation of translation preinitiation complex	4	2.56	0.000870674	8662, 8661	140	23	16792	20.86	0.48057458	0.03388787	1.31744302
GOTERM_CC_DIRECT	GO:0071541	eukaryotic translation initiation factor 3 complex, e	3	1.92	0.00132157	8662, 8661	148	7	18224	52.77	0.26615096	0.01535351	1.68572492
GOTERM_MF_DIRECT	GO:0003743	translation initiation factor activity	5	3.21	0.001474097	9470, 8662	137	61	16881	10.10	0.26855923	0.06063185	1.84930867
GOTERM_BP_DIRECT	GO:0006446	regulation of translational initiation	4	2.56	0.00324534	8662, 8661	140	36	16792	13.33	0.91322743	0.08359999	4.82866524
GOTERM_BP_DIRECT	GO:0006413	translational initiation	6	3.85	0.005685435	9470, 8662	140	137	16792	5.25	0.9862628	0.12917236	8.31478483
GOTERM_MF_DIRECT	GO:0031369	translation initiation factor binding	3	1.92	0.012239971	8662, 7286	137	21	16881	17.60	0.92653052	0.25181002	14.429874

Annotation Cluster 4 — Enrichment Score: 2.2774804592397127

Category	Term		Count	%	PValue	Genes	List Total	Pop Hits	Pop Total	Fold Enrichm	Bonferroni	Benjamini	FDR
GOTERM_CC_DIRECT	GO:0033565	ESCRT-0 complex	3	1.92	0.000192849	10254, 802	148	3	18224	123.14	0.04412781	0.00375386	0.24763798
GOTERM_BP_DIRECT	GO:0016197	endosomal transport	5	3.21	0.002178226	10254, 887	140	66	16792	9.09	0.80598365	0.0634876	3.26550849
GOTERM_CC_DIRECT	GO:0031901	early endosome membrane	5	3.21	0.014504528	399979, 20	148	116	18224	5.31	0.96725205	0.12781751	17.1245603

GOTERM_BP_DIRECT	GO:0006914	autophagy	5	3.21	0.024870216	9779, 1025	140	133	16792	4.51	0.99999999	0.36992999	31.8488962
GOTERM_BP_DIRECT	GO:0036258	multivesicular body assembly	3	1.92	0.027045418	10254, 802	140	31	16792	11.61	1	0.38090344	34.1271211

Annotation Cluster 5 Enrichment Score: 2.1743126331310445

Category	Term		Count	%	PValue	Genes	List Total	Pop Hits	Pop Total	Fold Enrichm	Bonferroni	Benjamini	FDR
GOTERM_BP_DIRECT	GO:0070911	global genome nucleotide-excision repair	5	3.21	0.000135044	7341, 8451	140	32	16792	18.74	0.09657293	0.00722803	0.20541007
GOTERM_BP_DIRECT	GO:0000717	nucleotide-excision repair, DNA duplex unwinding	4	2.56	0.000761703	8451, 845C	140	22	16792	21.81	0.4361794	0.03133302	1.15344862
GOTERM_BP_DIRECT	GO:0000715	nucleotide-excision repair, DNA damage recognitior	4	2.56	0.000870674	8451, 845C	140	23	16792	20.86	0.48057458	0.03388787	1.31744302
GOTERM_BP_DIRECT	GO:0006294	nucleotide-excision repair, preincision complex asse	4	2.56	0.001732438	8451, 845C	140	29	16792	16.54	0.72853528	0.05511524	2.60542093
GOTERM_BP_DIRECT	GO:0042769	DNA damage response, detection of DNA damage	4	2.56	0.00324534	8451, 845C	140	36	16792	13.33	0.91322743	0.08359999	4.82866524
GOTERM_BP_DIRECT	GO:0006296	nucleotide-excision repair, DNA incision, 5'-to lesior	4	2.56	0.0035105	8451, 845C	140	37	16792	12.97	0.928962	0.0871567	5.21340489
GOTERM_BP_DIRECT	GO:0033683	nucleotide-excision repair, DNA incision	4	2.56	0.003788536	8451, 845C	140	38	16792	12.63	0.94240902	0.09076011	5.61526719
GOTERM_BP_DIRECT	GO:0006293	nucleotide-excision repair, preincision complex stat	3	1.92	0.012888035	8451, 845C	140	21	16792	17.13	0.99994198	0.24323955	17.9218784
GOTERM_BP_DIRECT	GO:0006295	nucleotide-excision repair, DNA incision, 3'-to lesior	3	1.92	0.015360668	8451, 845C	140	23	16792	15.64	0.9999912	0.26386252	20.9970071
GOTERM_BP_DIRECT	GO:0006283	transcription-coupled nucleotide-excision repair	4	2.56	0.02347942	8451, 845C	140	74	16792	6.48	0.99999998	0.36024995	30.3538701
GOTERM_BP_DIRECT	GO:0042787	protein ubiquitination involved in ubiquitin-depend	5	3.21	0.038386209	5347, 8451	140	153	16792	3.92	1	0.46542219	44.8961999
GOTERM_MF_DIRECT	GO:0061630	ubiquitin protein ligase activity	4	2.56	0.191583872	8315, 8451	137	187	16881	2.64	1	0.84720509	93.2195215
GOTERM_BP_DIRECT	GO:0043161	proteasome-mediated ubiquitin-dependent proteir	4	2.56	0.236724205	8451, 9978	140	203	16792	2.36	1	0.93336745	98.3639403

Annotation Cluster 6 Enrichment Score: 1.953777794242805

Category	Term		Count	%	PValue	Genes	List Total	Pop Hits	Pop Total	Fold Enrichm	Bonferroni	Benjamini	FDR
GOTERM_BP_DIRECT	GO:0070423	nucleotide-binding oligomerization domain contain	5	3.21	4.9689E-05	23118, 104	140	25	16792	23.99	0.03667749	0.00339124	0.07562583
GOTERM_CC_DIRECT	GO:0010008	endosome membrane	8	5.13	0.000766167	9779, 2311	148	185	18224	5.32	0.1641882	0.00991451	0.9805006
GOTERM_BP_DIRECT	GO:0007179	transforming growth factor beta receptor signaling	6	3.85	0.000998721	9958, 4738	140	92	16792	7.82	0.5283006	0.03687365	1.50982109
GOTERM_BP_DIRECT	GO:0007249	I-kappaB kinase/NF-kappaB signaling	5	3.21	0.001531154	23118, 104	140	60	16792	10.00	0.68409379	0.05102965	2.30599846
GOTERM_BP_DIRECT	GO:0002755	MyD88-dependent toll-like receptor signaling pathv	4	2.56	0.002525136	23118, 104	140	33	16792	14.54	0.85062631	0.06799647	3.77629028
GOTERM_BP_DIRECT	GO:0007254	JNK cascade	4	2.56	0.007745364	23118, 104	140	49	16792	9.79	0.99711199	0.16237727	11.1645115
GOTERM_BP_DIRECT	GO:0002223	stimulatory C-type lectin receptor signaling pathwa	4	2.56	0.056566796	23118, 104	140	105	16792	4.57	1	0.56919134	58.7929391
GOTERM_BP_DIRECT	GO:0000187	activation of MAPK activity	4	2.56	0.059197228	23118, 104	140	107	16792	4.48	1	0.57929277	60.5079196
GOTERM_BP_DIRECT	GO:0051092	positive regulation of NF-kappaB transcription factc	4	2.56	0.099240686	23118, 104	140	133	16792	3.61	1	0.7360911	79.6338885
GOTERM_BP_DIRECT	GO:0043123	positive regulation of I-kappaB kinase/NF-kappaB si	4	2.56	0.149241293	23118, 688	140	161	16792	2.98	1	0.84586046	91.4633674
GOTERM_BP_DIRECT	GO:0038095	Fc-epsilon receptor signaling pathway	4	2.56	0.183253713	23118, 104	140	178	16792	2.70	1	0.89689568	95.413227
GOTERM_BP_DIRECT	GO:0050852	T cell receptor signaling pathway	3	1.92	0.346576489	23118, 688	140	148	16792	2.43	1	0.9807568	99.8464328

Annotation Cluster 7 Enrichment Score: 1.4814722409035246

Category	Term		Count	%	PValue	Genes	List Total	Pop Hits	Pop Total	Fold Enrichm	Bonferroni	Benjamini	FDR
GOTERM_CC_DIRECT	GO:0005913	cell-cell adherens junction	8	5.13	0.015957793	117178, 26	148	323	18224	3.05	0.97681474	0.134786	18.6820284
GOTERM_MF_DIRECT	GO:0098641	cadherin binding involved in cell-cell adhesion	7	4.49	0.030094909	26058, 913	137	290	16881	2.97	0.99846327	0.39244652	32.067342
GOTERM_BP_DIRECT	GO:0098609	cell-cell adhesion	6	3.85	0.074837179	26058, 913	140	271	16792	2.66	1	0.66150149	69.4040448

Annotation Cluster 8 Enrichment Score: 1.4668518040035676

Category	Term		Count	%	PValue	Genes	List Total	Pop Hits	Pop Total	Fold Enrichm	Bonferroni	Benjamini	FDR
GOTERM_BP_DIRECT	GO:0035735	intraciliary transport involved in cilium morphogen	3	1.92	0.001390288	117178, 51	140	7	16792	51.40	0.6487385	0.04859953	2.09594166
GOTERM_BP_DIRECT	GO:0060271	cilium morphogenesis	4	2.56	0.104362232	117178, 51	140	136	16792	3.53	1	0.748777	81.3273939
GOTERM_BP_DIRECT	GO:0042384	cilium assembly	3	1.92	0.274028878	117178, 51	140	124	16792	2.90	1	0.95256564	99.2371036

Annotation Cluster 9 Enrichment Score: 1.0790518054724672

Category	Term		Count	%	PValue	Genes	List Total	Pop Hits	Pop Total	Fold Enrichm	Bonferroni	Benjamini	FDR
GOTERM_MF_DIRECT	GO:0004843	thiol-dependent ubiquitin-specific protease activity	4	2.56	0.026794823	9958, 7353	137	80	16881	6.16	0.99684256	0.38111315	29.0839281

GOTERM_MF_DIRECT	GO:0036459	thiol-dependent ubiquitinyl hydrolase activity	3	1.92	0.109311533	9958, 8239	137	70	16881	5.28	1	0.68920768	76.8869921
GOTERM_BP_DIRECT	GO:0016579	protein deubiquitination	3	1.92	0.197755109	9958, 8239	140	99	16792	3.63	1	0.90940911	96.5081803

Annotation Cluster 10 Enrichment Score: 0.7601816526828035

Category	Term		Count	%	PValue	Genes	List Total	Pop Hits	Pop Total	Fold Enrichm	Bonferroni	Benjamini	FDR
GOTERM_BP_DIRECT	GO:0018105	peptidyl-serine phosphorylation	4	2.56	0.08533973	1020, 5347	140	125	16792	3.84	1	0.69175118	74.2858651
GOTERM_MF_DIRECT	GO:0004674	protein serine/threonine kinase activity	6	3.85	0.187108687	1020, 5347	137	376	16881	1.97	1	0.8518389	92.728948
GOTERM_MF_DIRECT	GO:0004672	protein kinase activity	5	3.21	0.32825385	1020, 5347	137	359	16881	1.72	1	0.94544804	99.3490567

Annotation Cluster 11 Enrichment Score: 0.735256209049127

Category	Term		Count	%	PValue	Genes	List Total	Pop Hits	Pop Total	Fold Enrichm	Bonferroni	Benjamini	FDR
GOTERM_CC_DIRECT	GO:0005667	transcription factor complex	6	3.85	0.020210022	7027, 1876	148	193	18224	3.83	0.99158372	0.15686493	23.0854708
GOTERM_MF_DIRECT	GO:0046983	protein dimerization activity	5	3.21	0.033174901	23269, 187	137	150	16881	4.11	0.99921701	0.37924931	34.7470641
GOTERM_MF_DIRECT	GO:0003677	DNA binding	14	8.97	0.598099143	84108, 230	137	1674	16881	1.03	1	0.99599971	99.9990211
GOTERM_BP_DIRECT	GO:0006351	transcription, DNA-templated	16	10.26	0.662541159	84108, 109	140	1955	16792	0.98	1	0.99986077	99.9999934
GOTERM_MF_DIRECT	GO:0003700	transcription factor activity, sequence-specific DNA	7	4.49	0.793152102	84108, 702	137	961	16881	0.90	1	0.99980948	99.9999998

Supplementary Figure S1

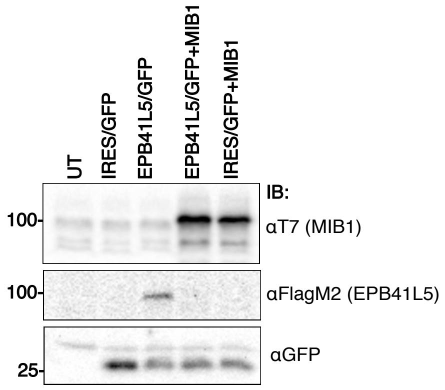

Figure S1. Expression of Flag-EPB41L5/IRES/GFP and T7-MIB1 in Hela cells.
Representative immunoblots showing the relative expression of transfected proteins. Equal cell numbers from the cell preparations depicted in Figure 5 were lysed in 2xLaemmli sample buffer and immunoblotted for the transfected proteins as indicated.

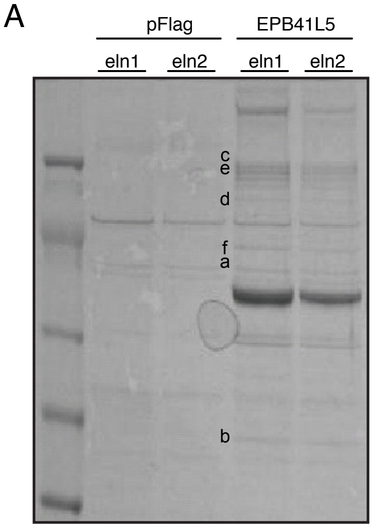

B

Bait	Common Name of Prey	No. of Peptides	Protein GI
EPB41L5	a. Mindbomb (MIB1)	12	30348954
	b. NaK Beta 3 subunit	1	15080119
	c. Zona Occludens 1 (ZO-1)	13	62945799
	d. Zona Occludens 2 (ZO-2)	19	13634076
	e. Crumbs1 (CRB1)	2	6912322
	f. Pinin	18	33356174

Figure S2. Identification of EPB41L5 interacting proteins. (A) Representative image of a colloidal Coomassie stained gel used in the identification of EPB41L5 binding partners by LC MS/MS. HEK293T cell lines stably expressing either pFLAG or pFLAG-EPB41L5 were lysed and immunoprecipitated with anti-FLAG M2 affinity gel. Proteins were eluted from the matrix using a 3X FLAG peptide and resolved by NuPage gel electrophoresis. Bands corresponding to proteins unique to the EPB41L5 elutions were excised, processed and subjected to LC MS/MS. (B) A representative summary of the prevalent proteins identified by the mass spectrometer is listed and denoted on the gel. Eln, elution.

Mass Spectrometry Protein Sample Preparation: HEK293T stable cell lines over expressing FLAG-EPB41L5 were used in mass spectrometry experiments to identify novel EPB41L5 binding partners. Sample preparation including cell lysis, immunoprecipitation and mass spectrometry (LC-MS/MS) was performed as previously described (Chen and Gingras, 2007). Briefly, 18 150 mm tissue culture plates of 90% confluent cells expressing either pFLAG empty vector or pFLAG-EPB41L5 were lysed in MS lysis buffer [50 mM HEPES-KOH pH 7.5, 150 mM NaCl, 1mM EGTA, 10% glycerol, 0.5% Triton-X100, 100 M $ZnCl_2$, 1 mM Na3VO4, 20 mM β-glycerolphosphate, 10 mM NaF, 1 mM DTT, 10mM $Na_4P_2O_7$ supplemented with 1 mM PMSF, 1mM MgCl2, and 1 COMPLETE protease inhibitor tablet (Roche)], and subjected to two freeze-thaw cycles. Lysates were precleared with sepharose beads, and subjected to anti-FLAG immunoprecipitation (FLAG M2-agarose, Sigma) to isolate EPB41L5 interacting proteins. Immunoaffinity purified proteins were then eluted using 3XFLAG peptide and separated by SDS-PAGE using the NuPage system on a 4-12% gradient gel. The gel was fixed in 50% methanol/7% Acetic Acid and stained with GelCode Blue (Pierce). Gel bands were excised and The Mass Spectrometry Facility/Advanced Protein Technology Centre at The Hospital for Sick Children processed samples and performed LC-MS/MS analyses.

Supplementary Figure S3

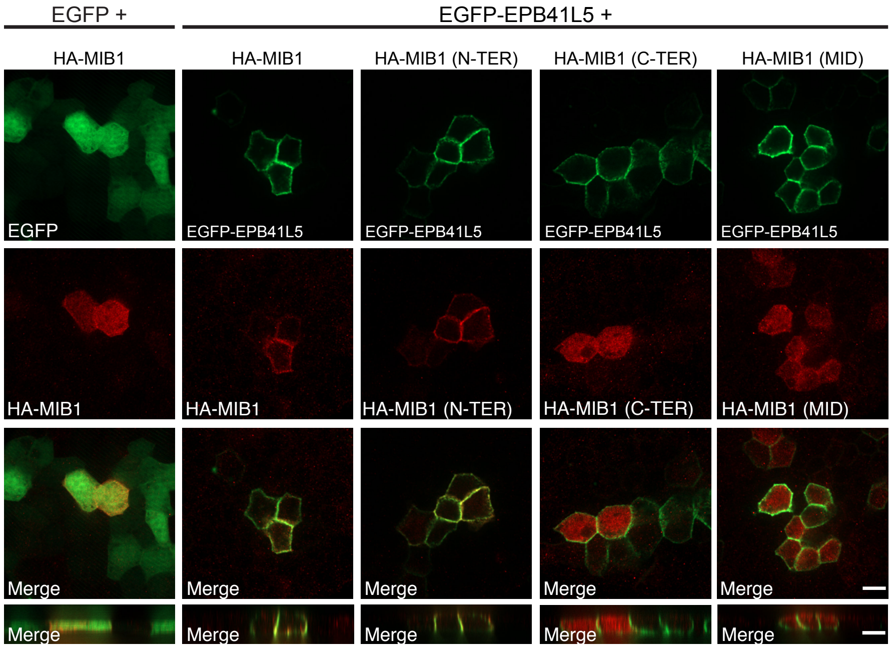

Figure S3. MIB1 relocalization to the plasma membrane when co-expressed with EPB41L5 requires the EPB41L5 interaction region. Polarized MDCK cells co-transfected with EGFP and HA-MIB1, EGFP-EPB41L5 and HA-MIB1, EGFP-EPB41L5 and HA-MIB1(N-Ter), EGFP-EPB41L5 and HA-MIB1(C-Ter), EGFP-EPB41L5 and HA-MIB1(Mid) where fixed and stained for HA (red). Shown are XY sections taken across the middle of the cell monolayer and XZ sections of the merge (bottom). Images were acquired at 63X magnification on a Quorum spinning disk microscope. Scale bars indicate 10 μm.

Supplementary Figure S4: Original western blots showing cropped regions used to prepare figures in the main text.

a. Figure 2a, b and c original blots
b. Figure 2d and 2e original blots
c. Figure 3a and 3c original blots
d. Figure 3d original blots
e. Figure 3e original blots
f. Figure 3f original blots
g. Figure 4a 3b and 4b original blots
h. Figure 4c original blots
i. Figure 6b original blots
j. Figure 6c original blots
k. Figure 6e original blots
l. Figure 7a
m. Figure 7b

FIGURE 2A, 2B and 2C original blots

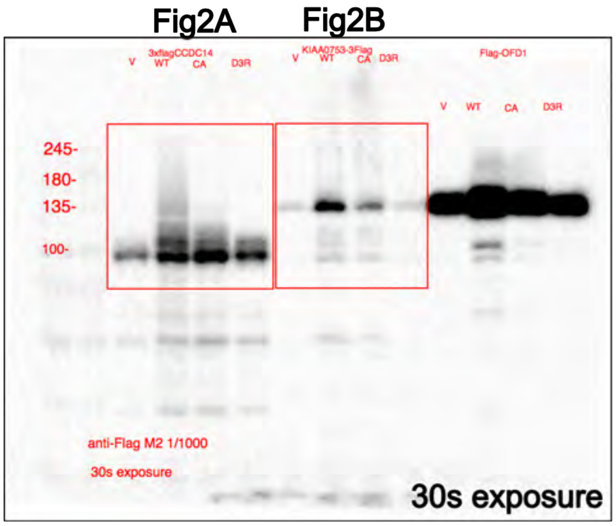

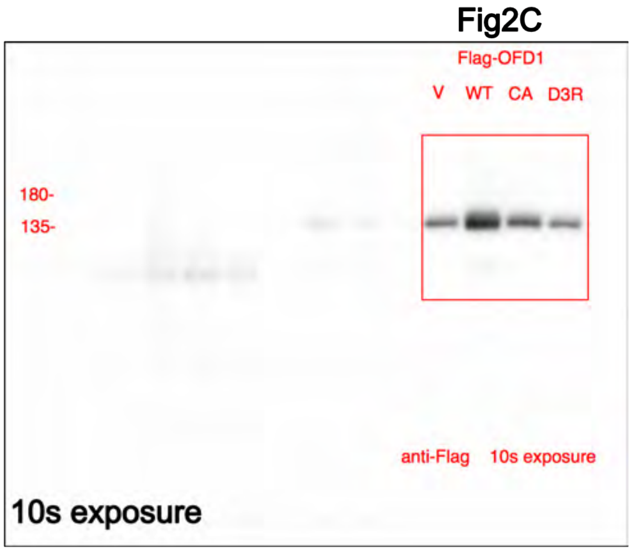

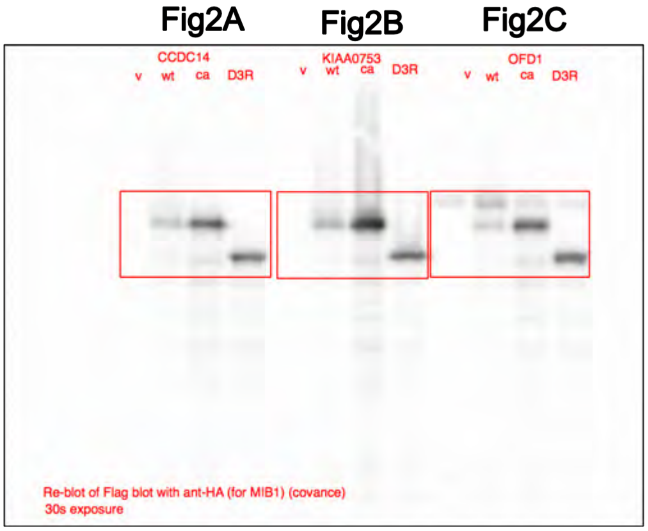

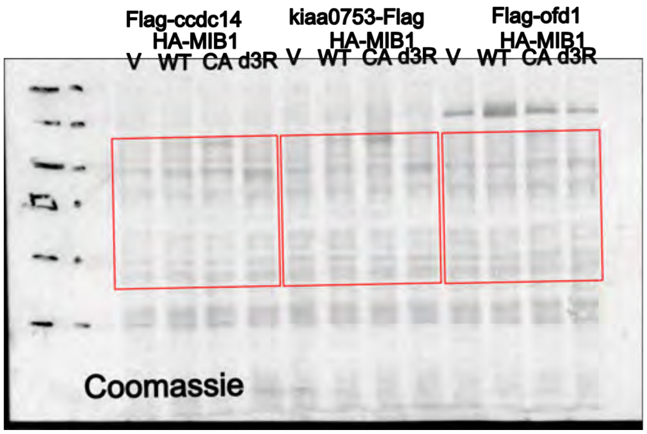

FIGURE 2D and 2E original blots

Fig 2D

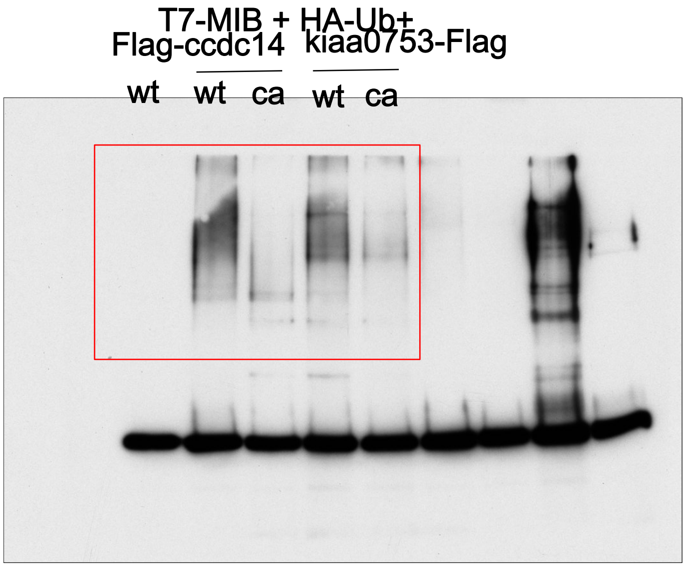

Fig 2E

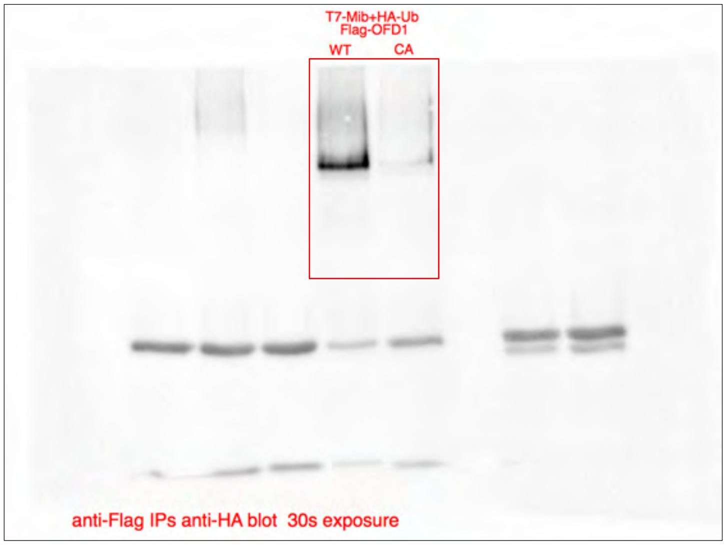

Fig 2D

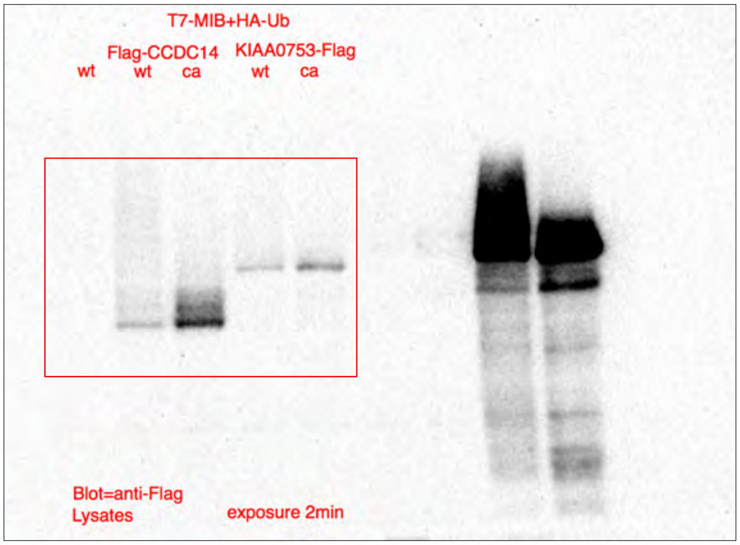

Fig 2E

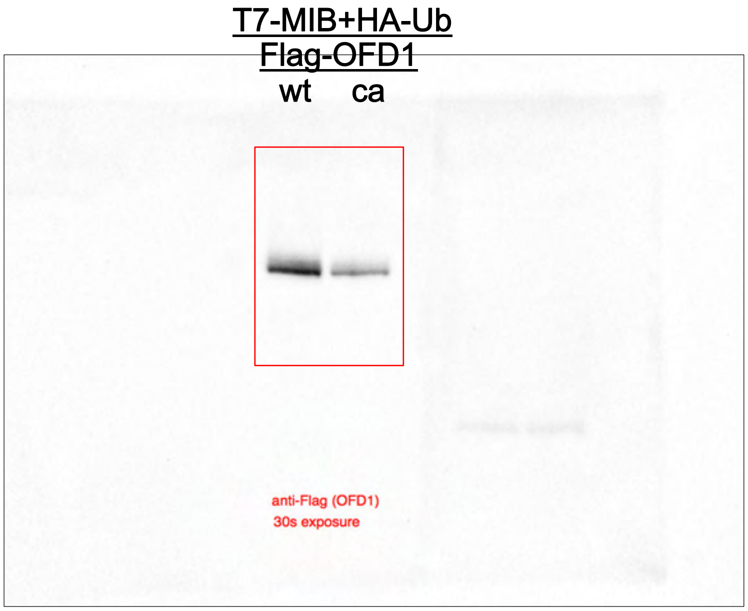

Fig 2D

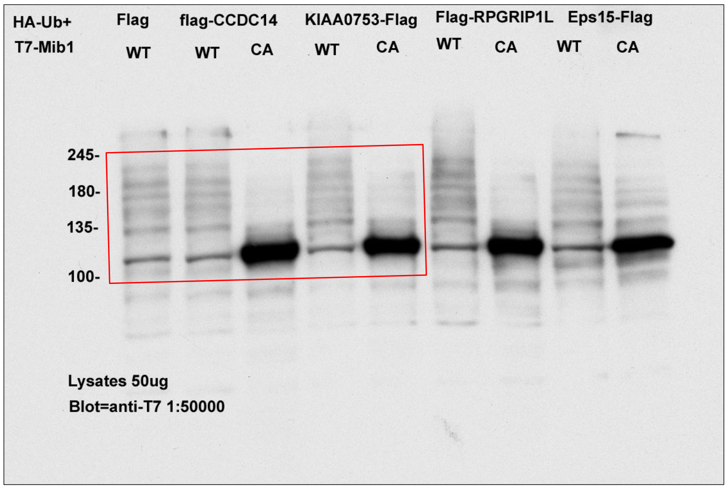

Fig 2E

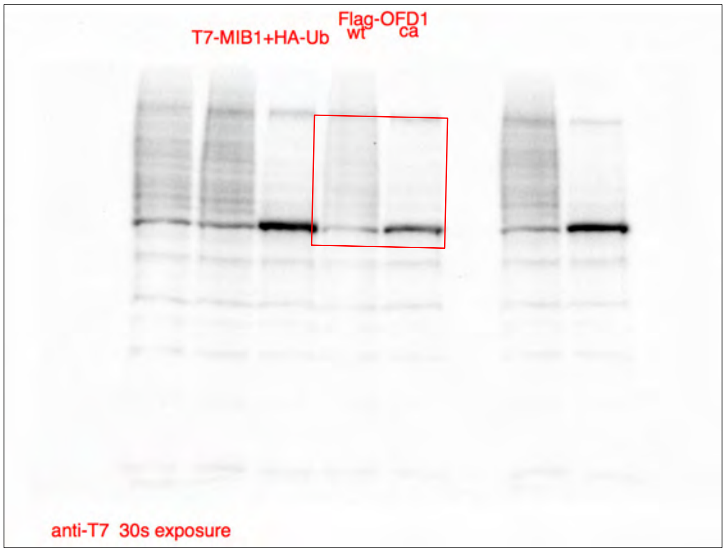

FIGURE 3A and 3C original blots

Fig 3A

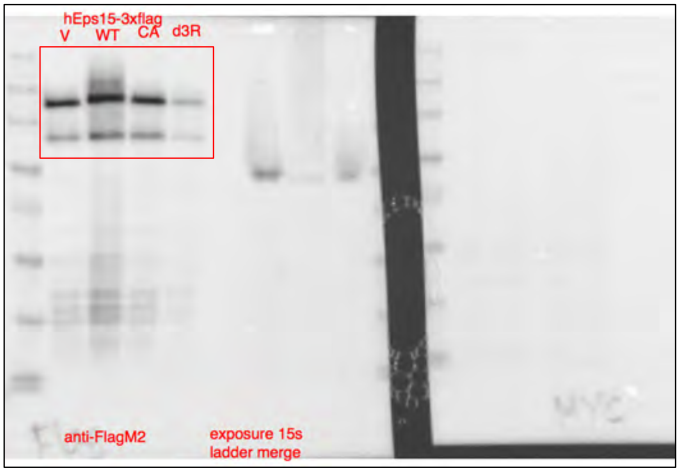

Fig 3C

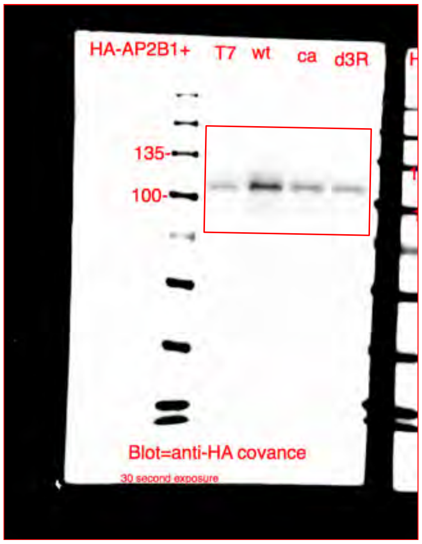

Fig 3A

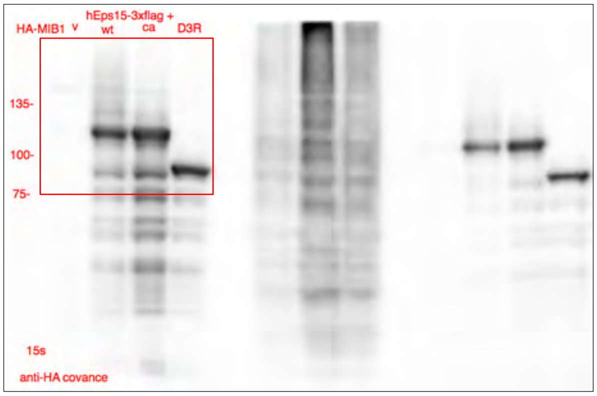

Fig 3C

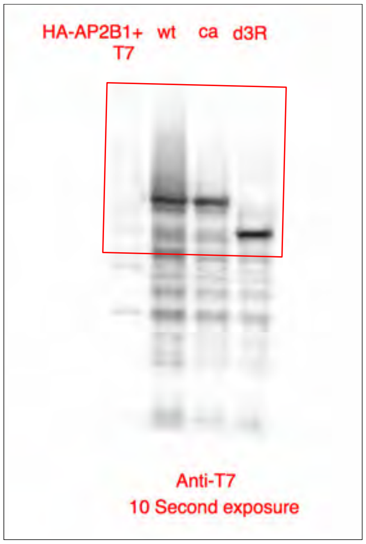

Fig 3A

hEps15-Flag
HA-Mib
v wt ca d3R

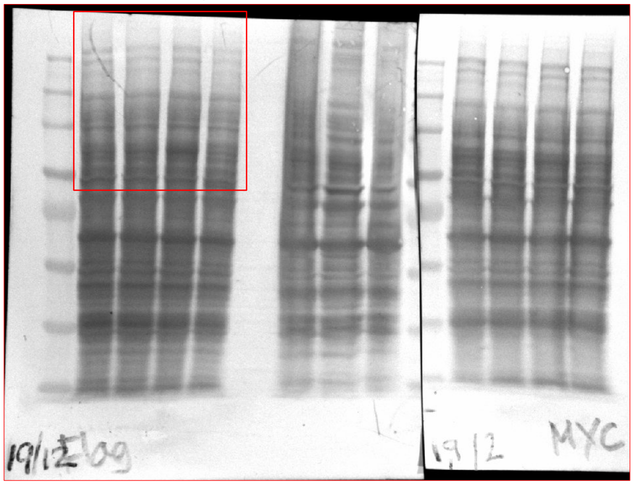

Fig 3C

HA-AP2B1
T7-Mib
v wt ca d3R

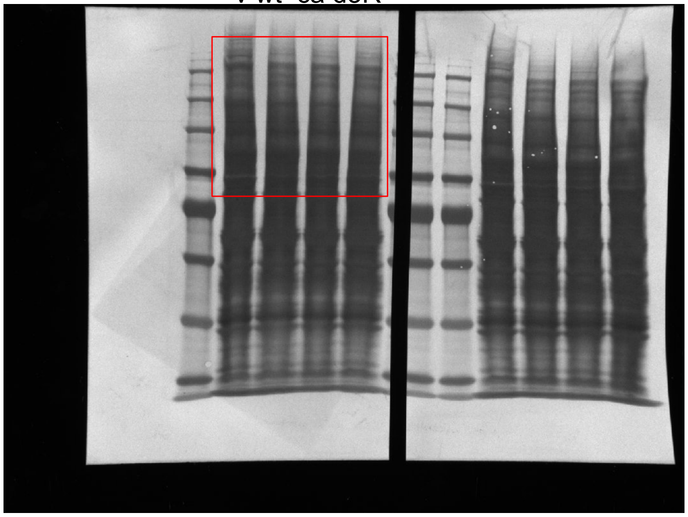

FIGURE 3D original blots

Fig 3D

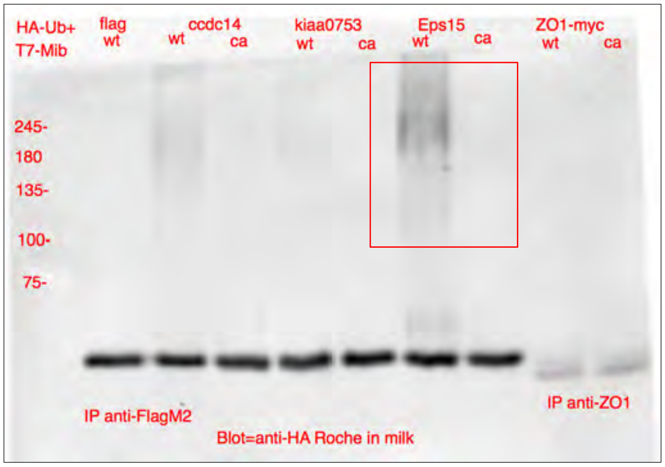

Fig 3D

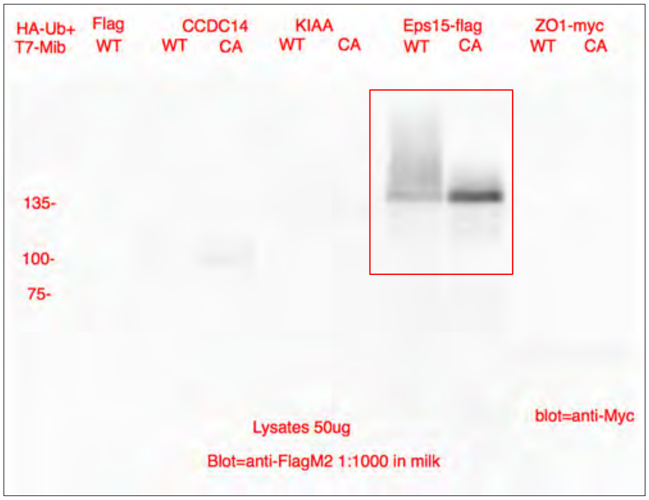

Fig 3D

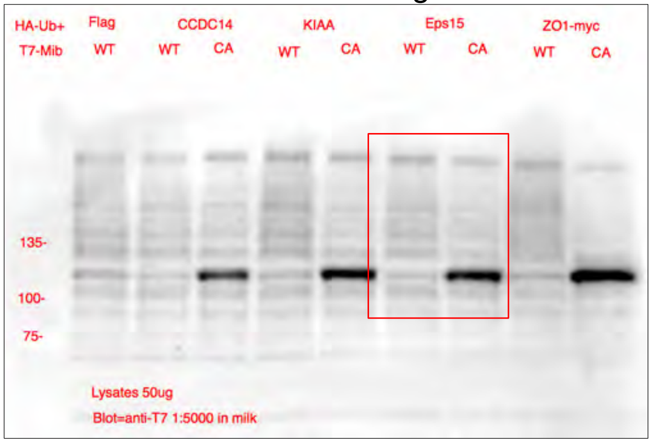

Figure 3e Original Blots

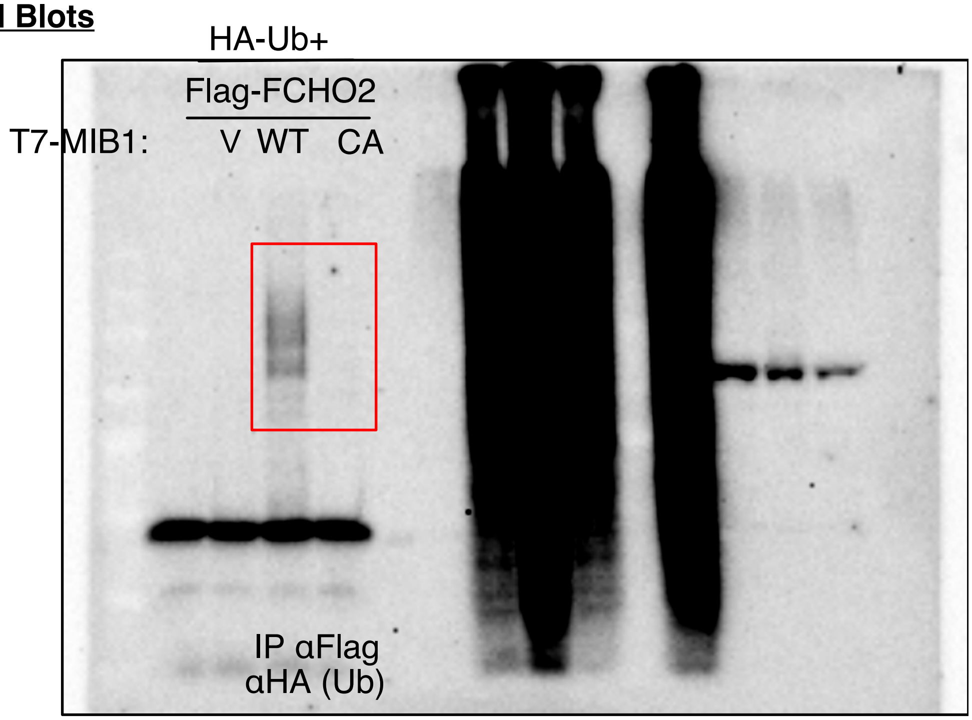

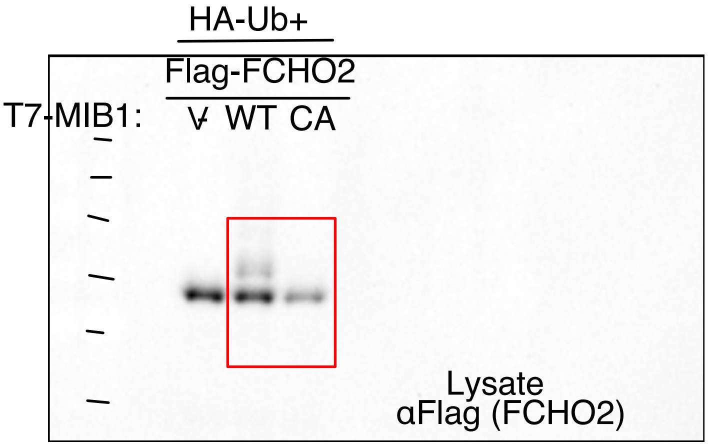

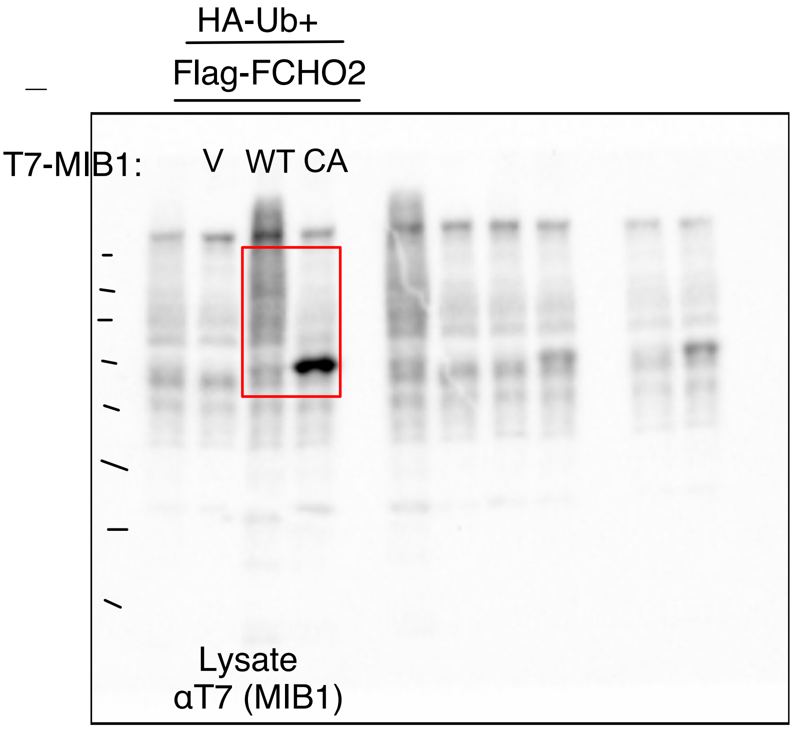

Figure 3f original blots

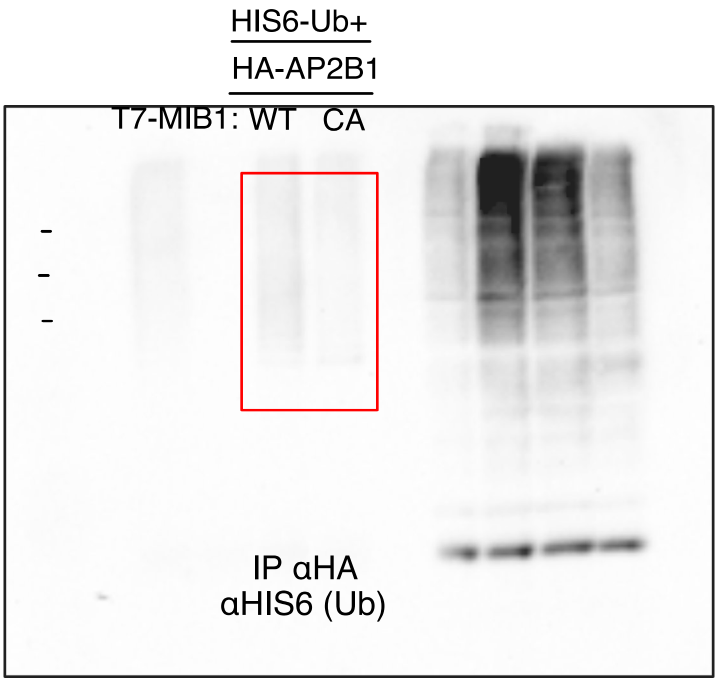

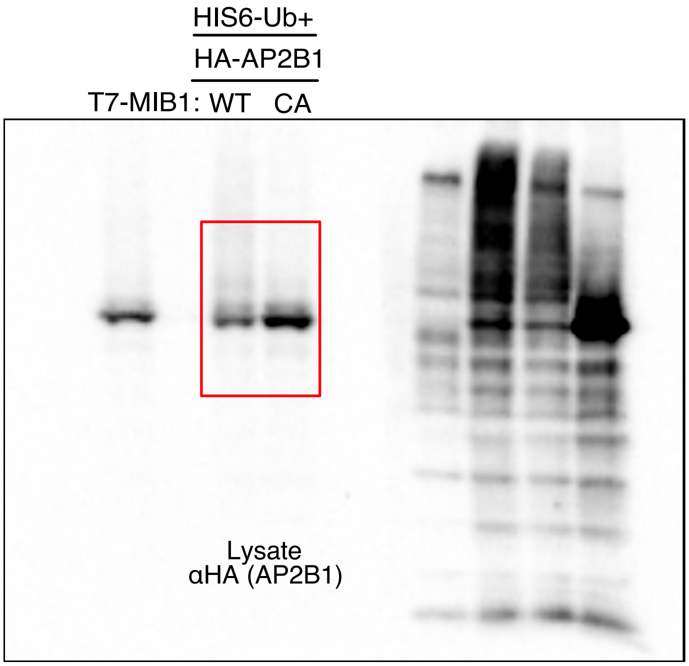

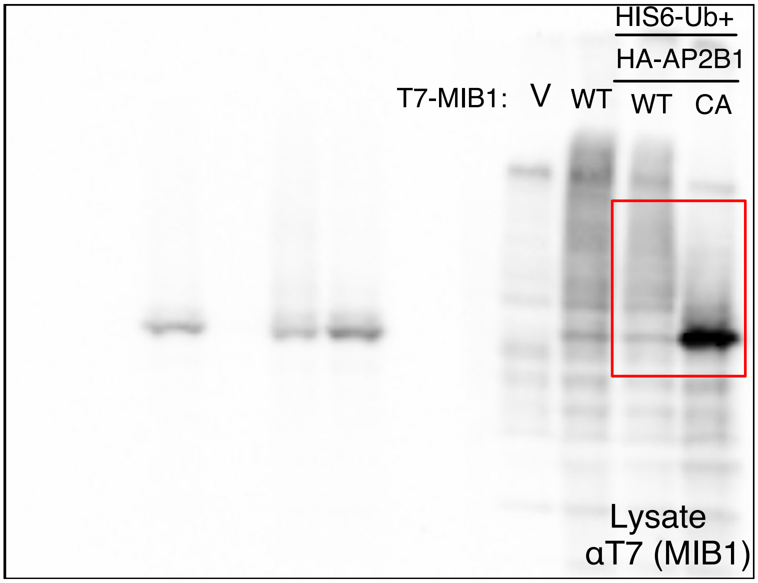

FIGURE 3B, 4A and 4B original blots

Fig 4A

Fig 3B

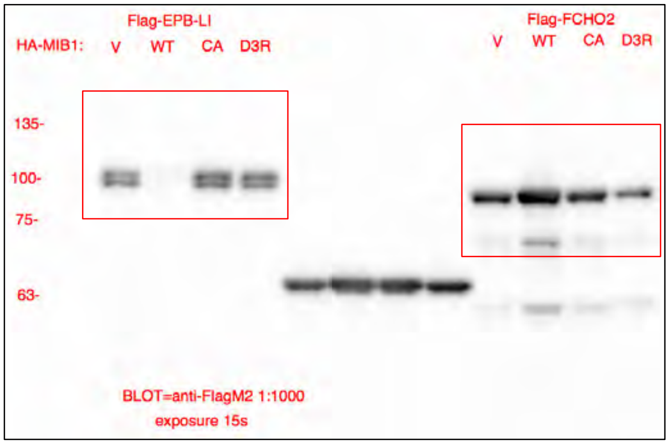

Fig 4B

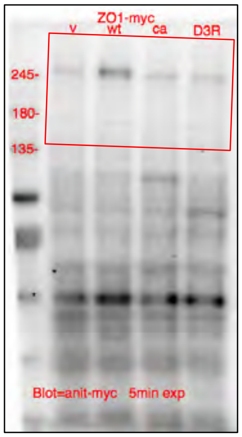

Fig 4A

Flag-EPB41L5

T7-Mib

v wt ca d3R

Fig 3B

Flag-FCHO2

T7-Mib

v wt ca d3R

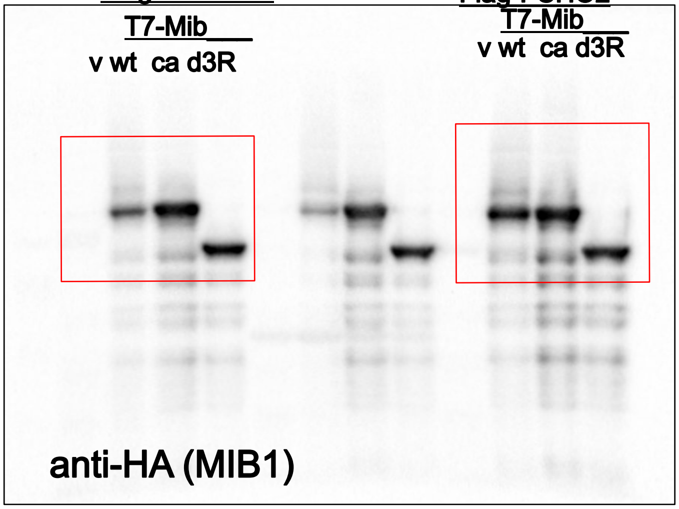

Fig 4B

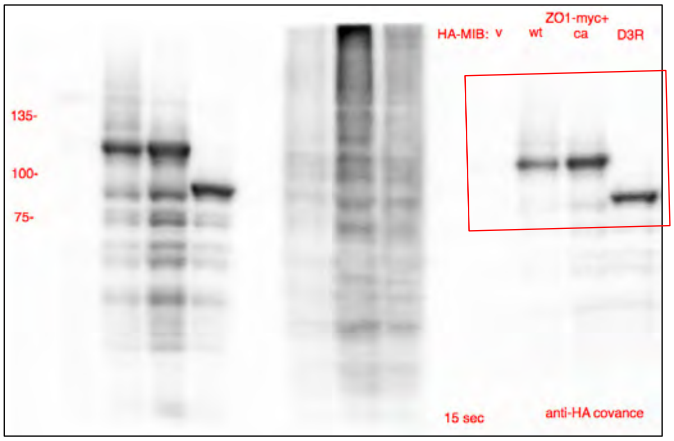

Fig 4A

Flag-EPB41L5

T7-Mib

v wt ca d3R

Fig 3B

Flag-FCHO2

T7-Mib

v wt ca d3R

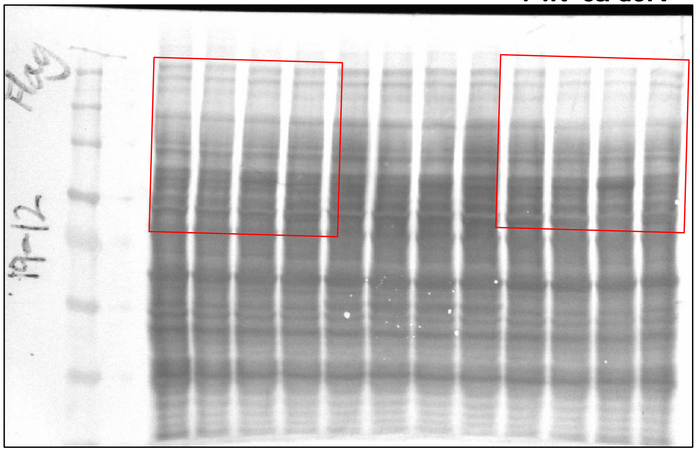

Coomassie

Fig 4B

ZO1-myc

HA-Mib

v wt ca d3R

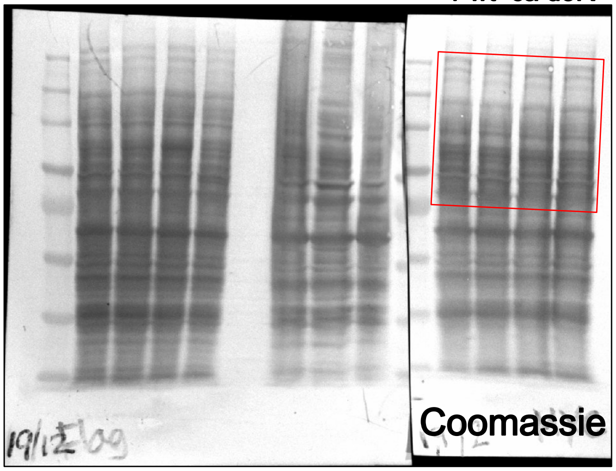

FIGURE 4C original blots

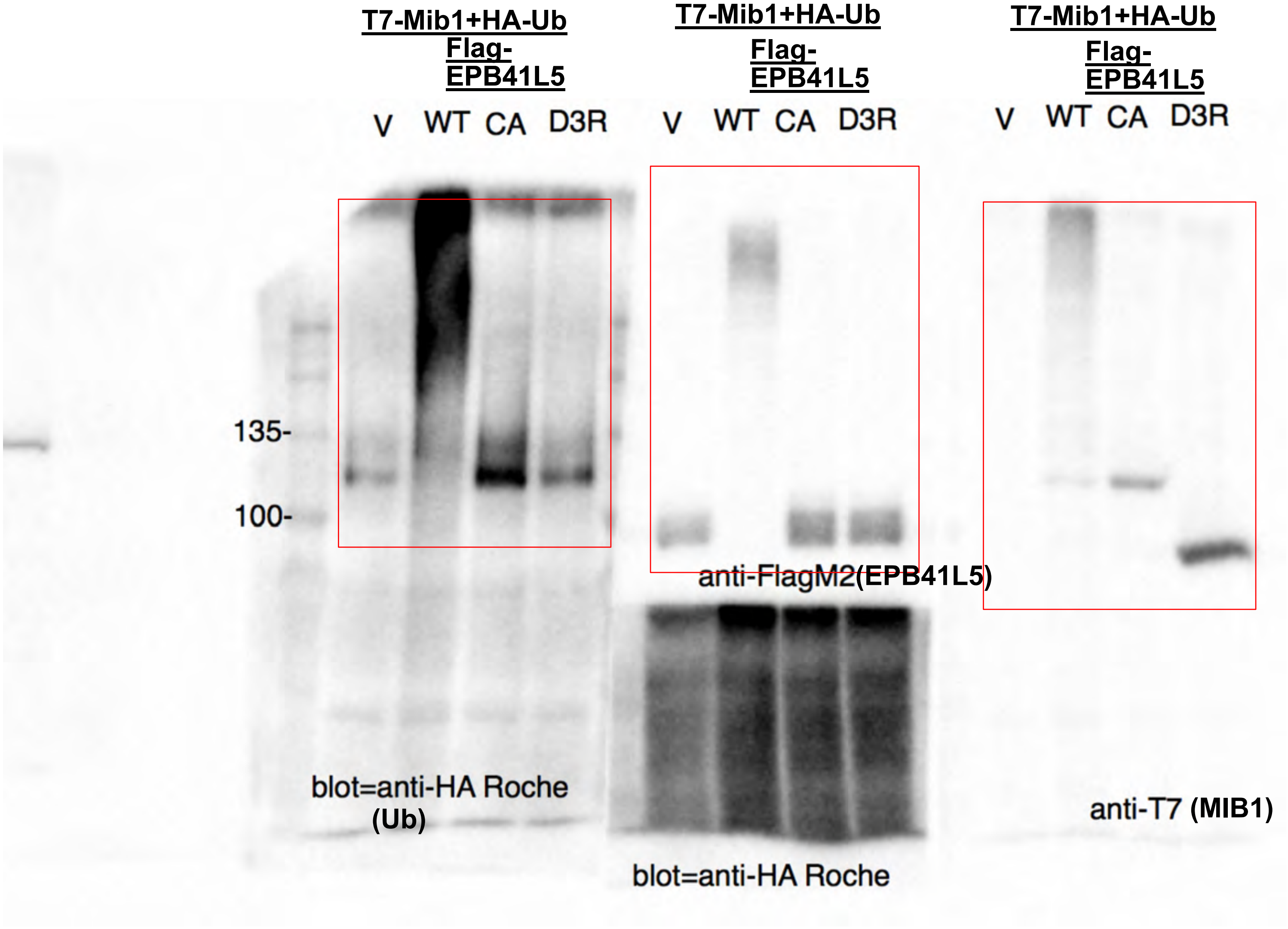

Figure 6B original blots

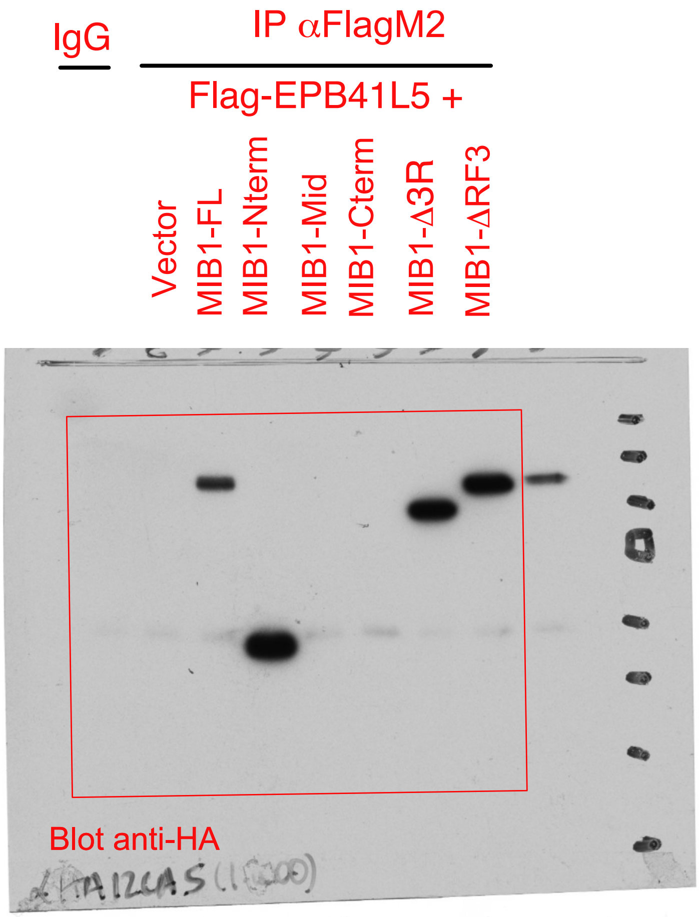

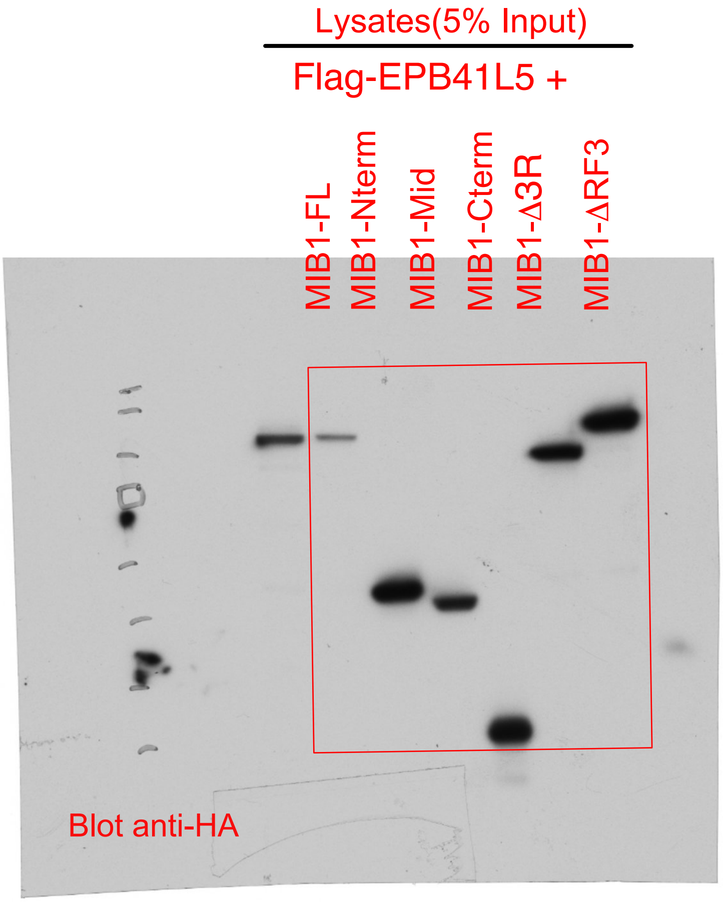

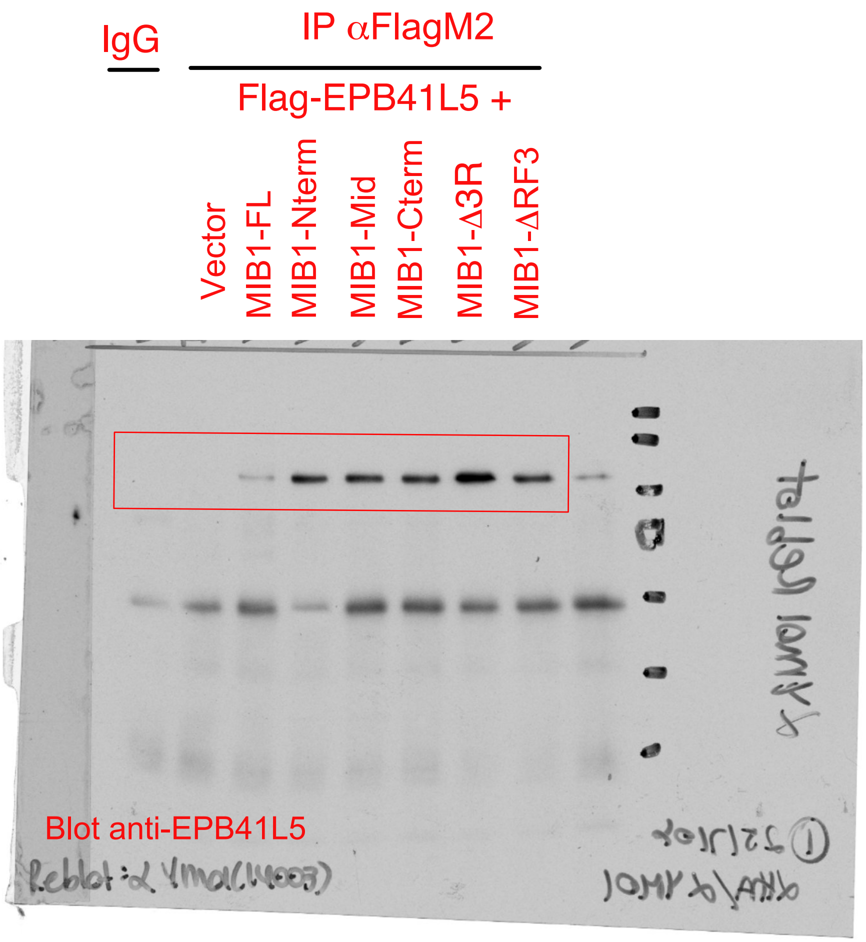

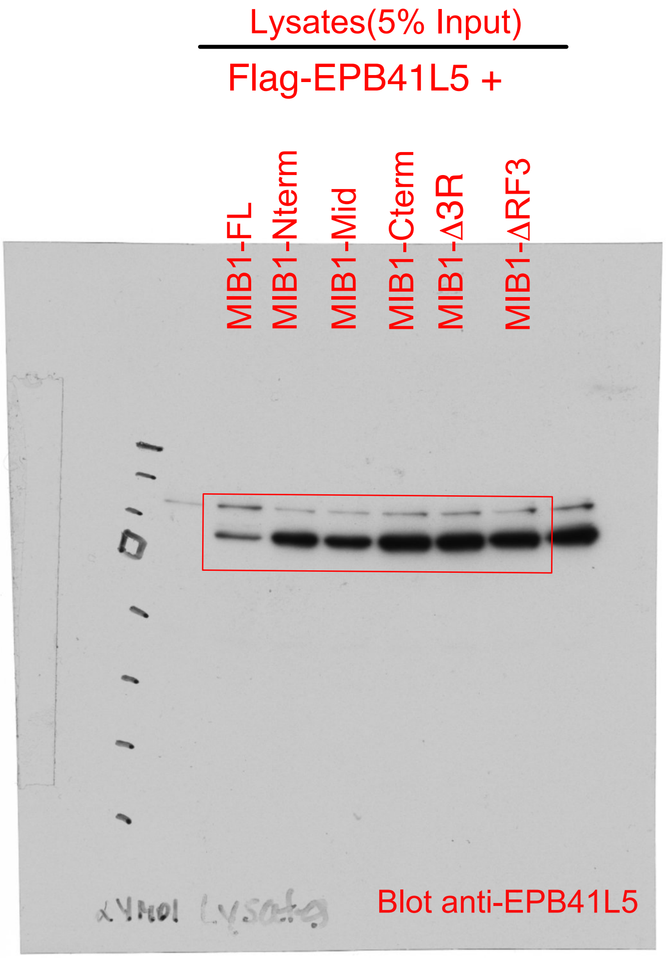

Figure 6C original blots

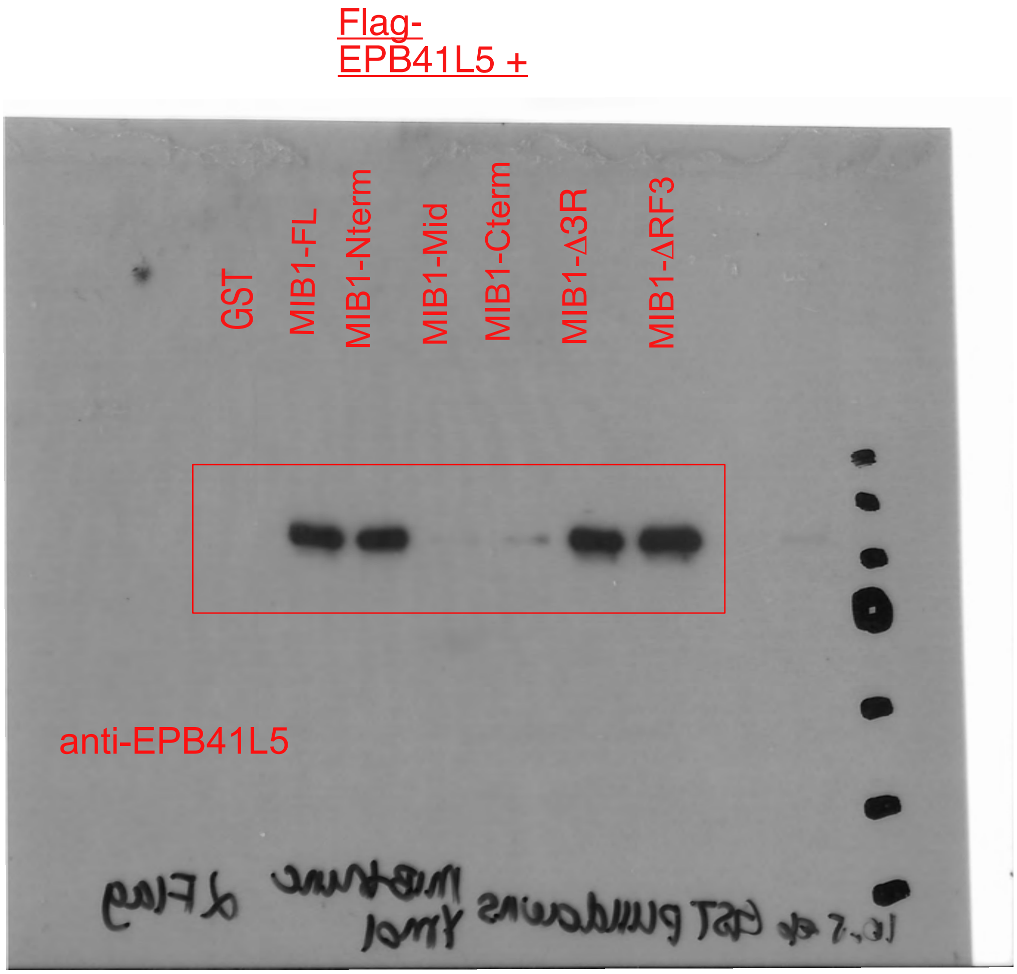

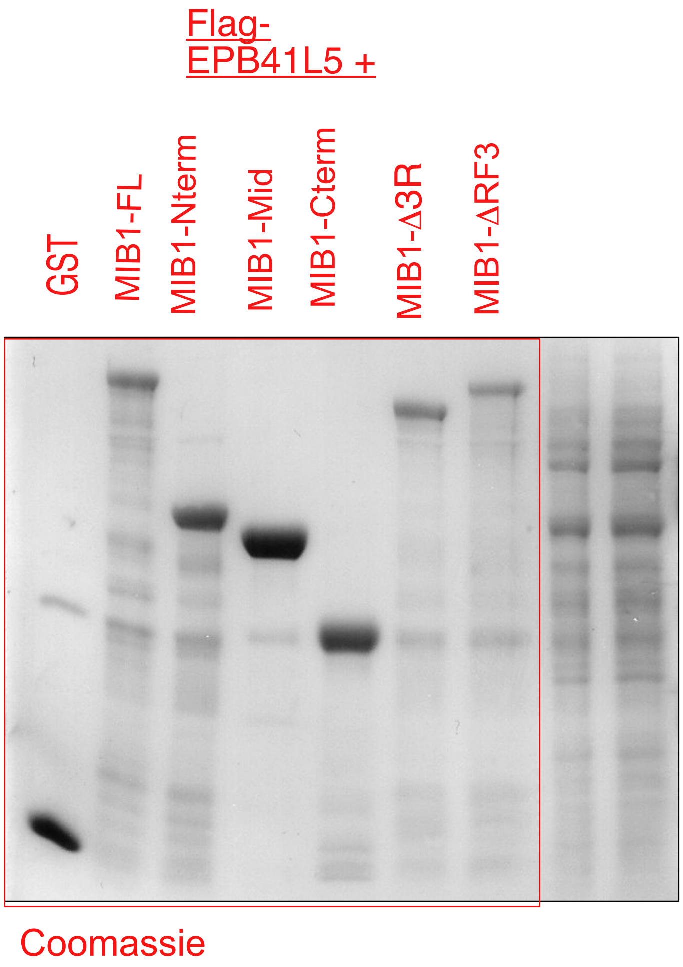

Figure 6E original blots

1-Vector

2- EPB41L5 + MIB1wt

3- EPB41L5

4- EPB41L5ΔPDB

5- EPB41L5 (1-660)

6- EPB41L5Nterm+FERM/FA

7- EPB41L5 (240-733)

8- EPB41L5 (333-733)

9- EPB41L5ΔFA

10- EPB41L5ΔFERM/FA

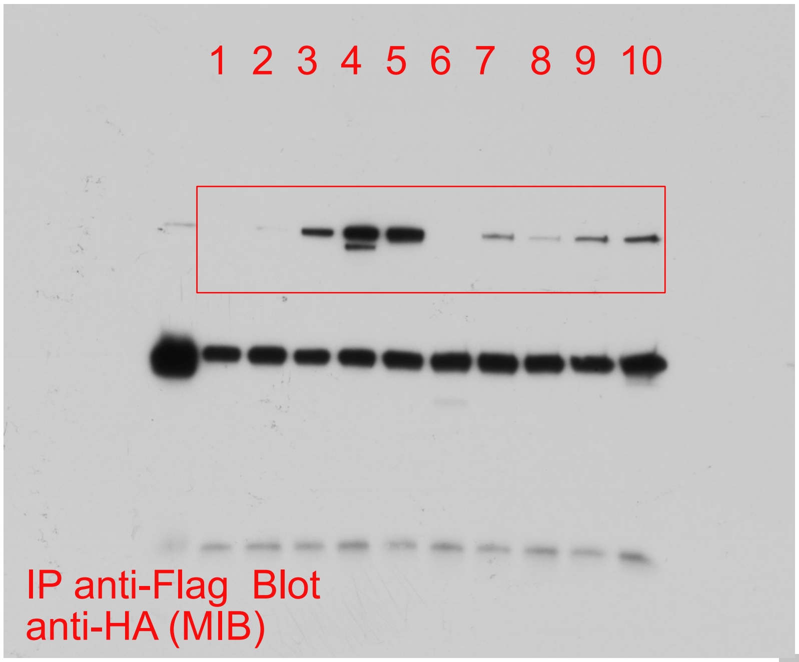

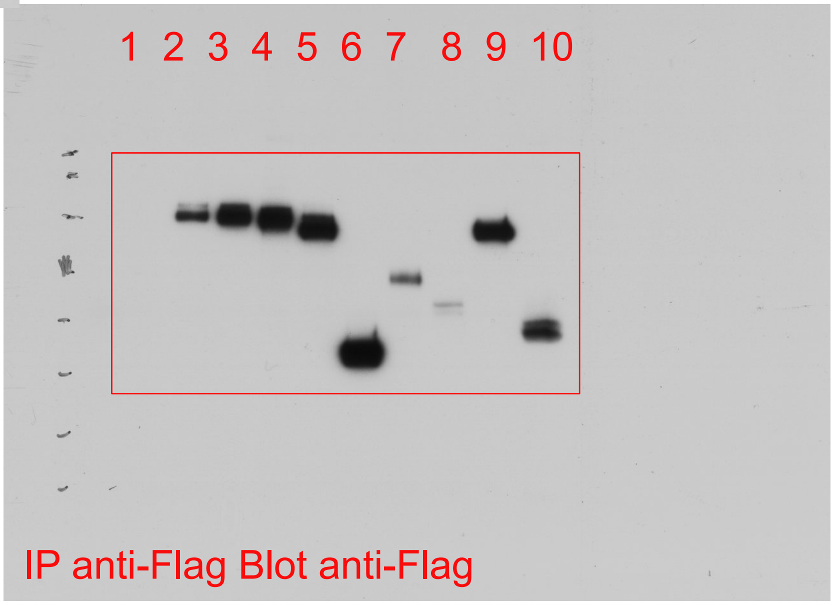

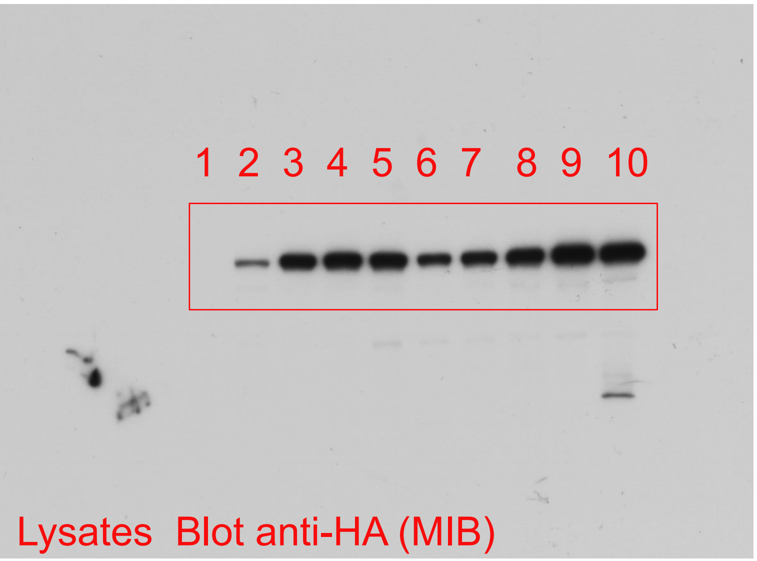

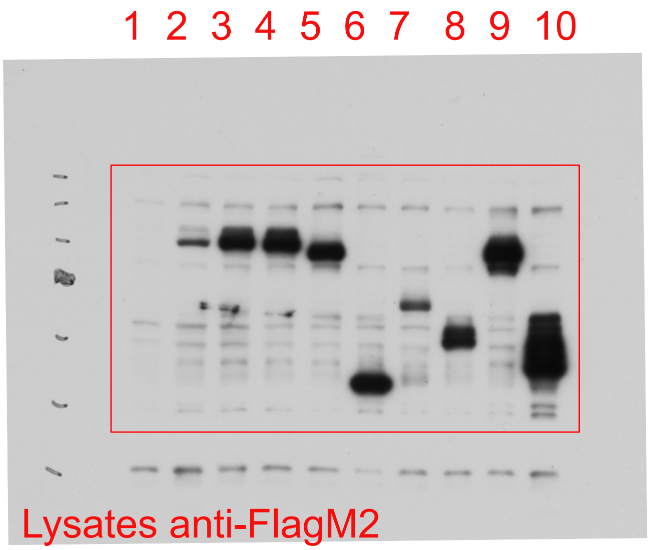

Figure 7A original blots

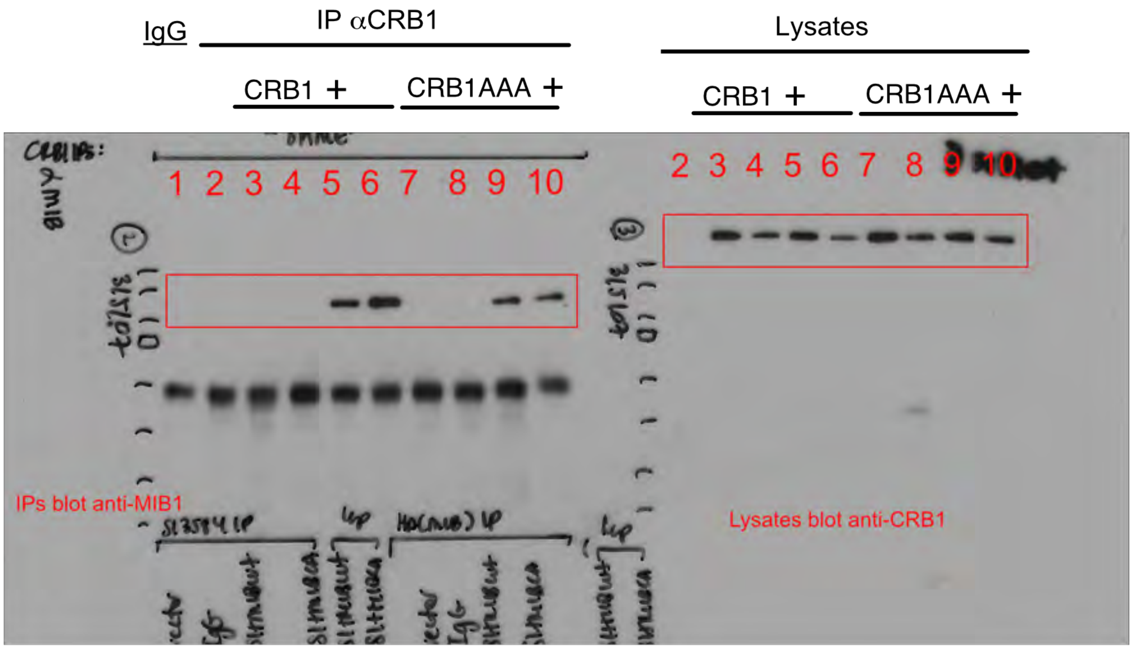

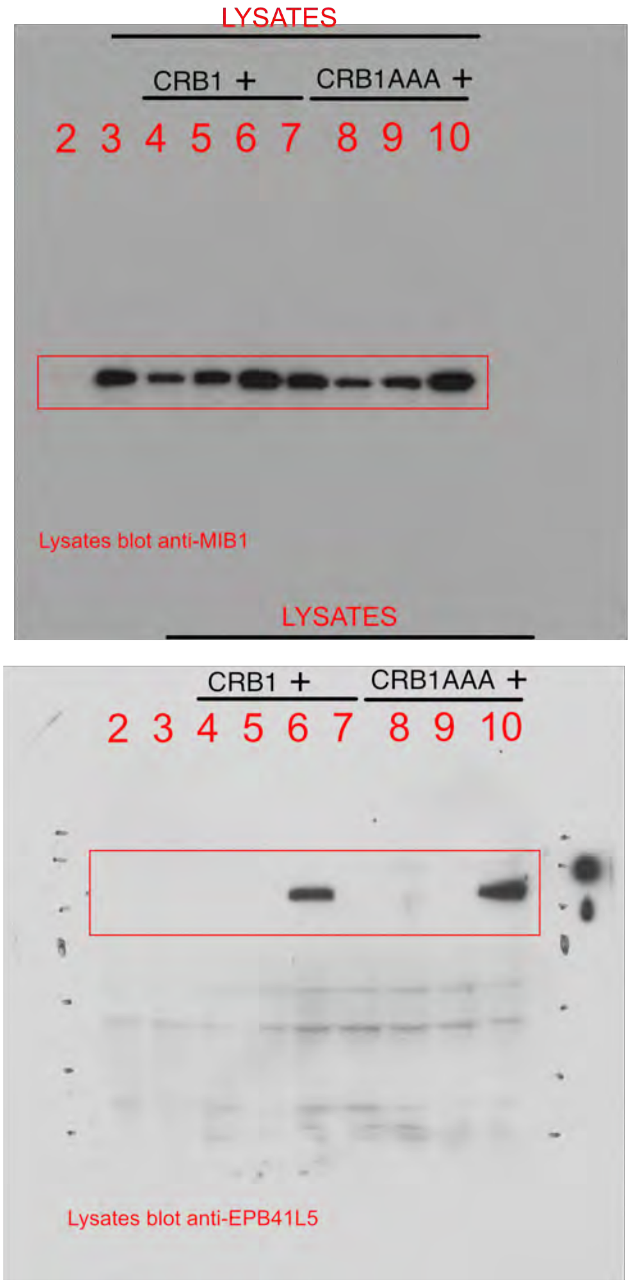

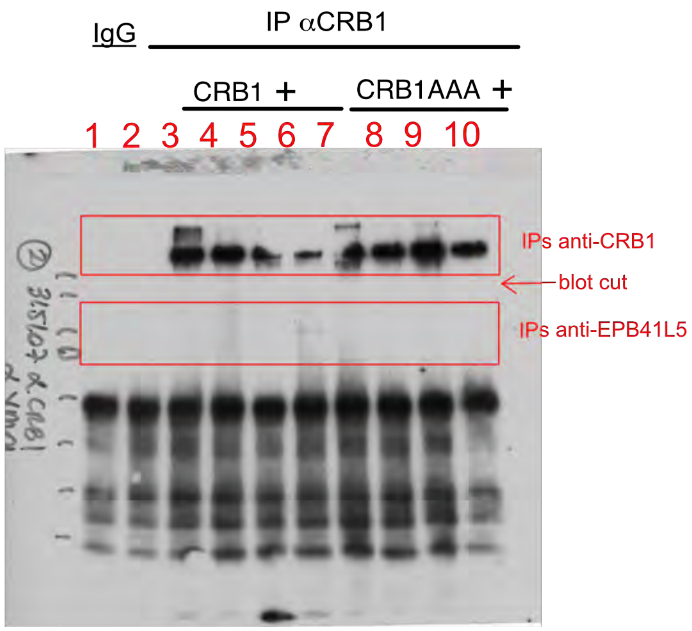

1- CRB1 + MIB1wt + EPB41L5
2- Vector
3- MIB1wt
4- MIB1wt + EPB41L5
5- MIB1CA
6- MIB1CA + EPB41L5
7- MIB1wt
8- MIB1wt + EPB41L5
9- MIB1CA
10- MIB1CA + EPB41L5

Figure 7B original blots

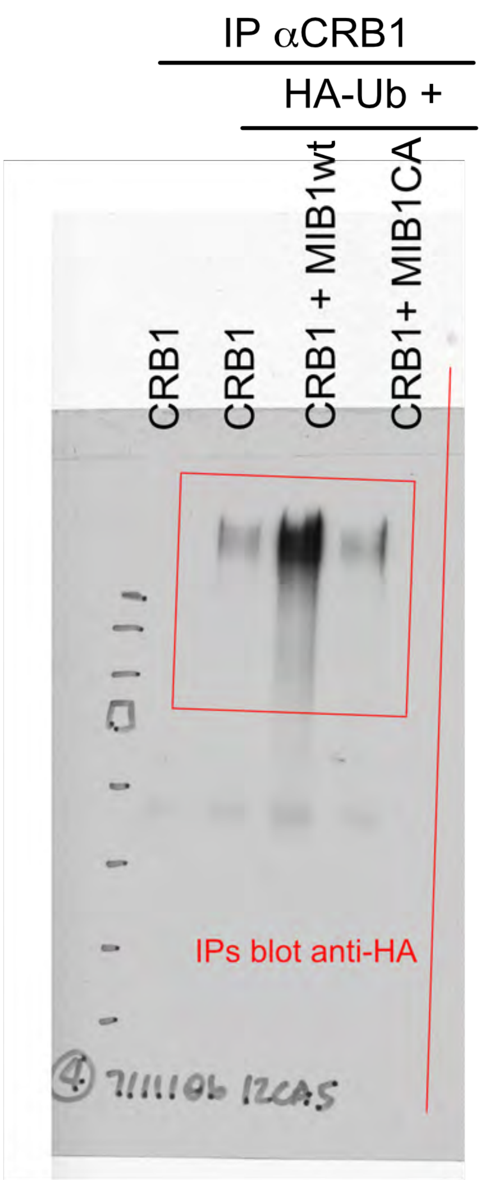

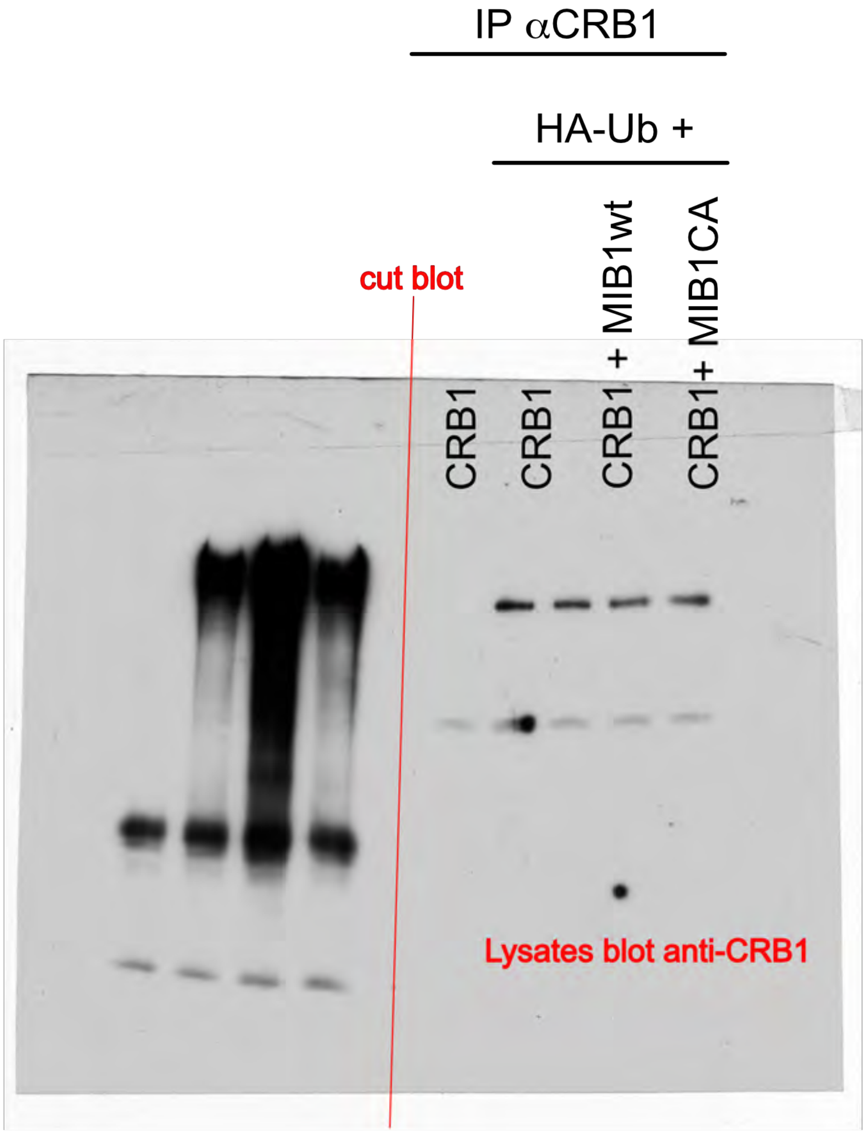

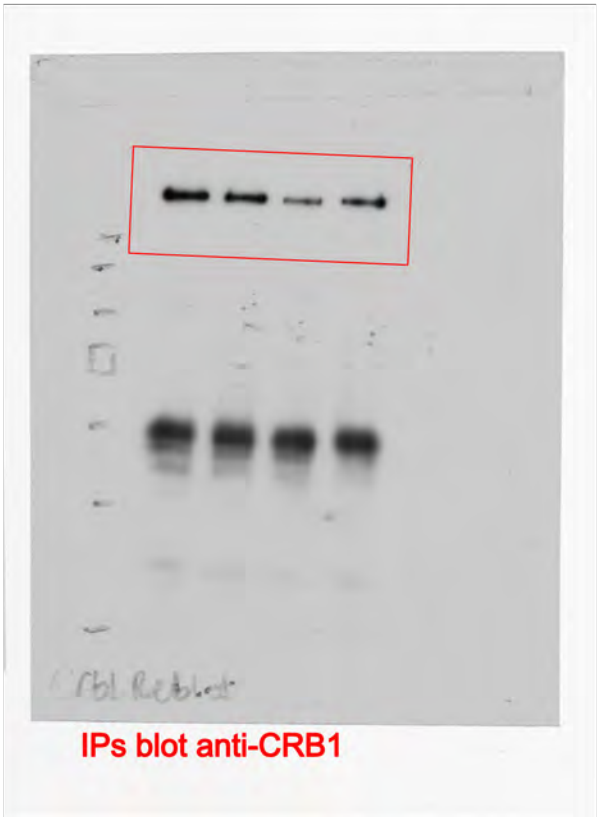

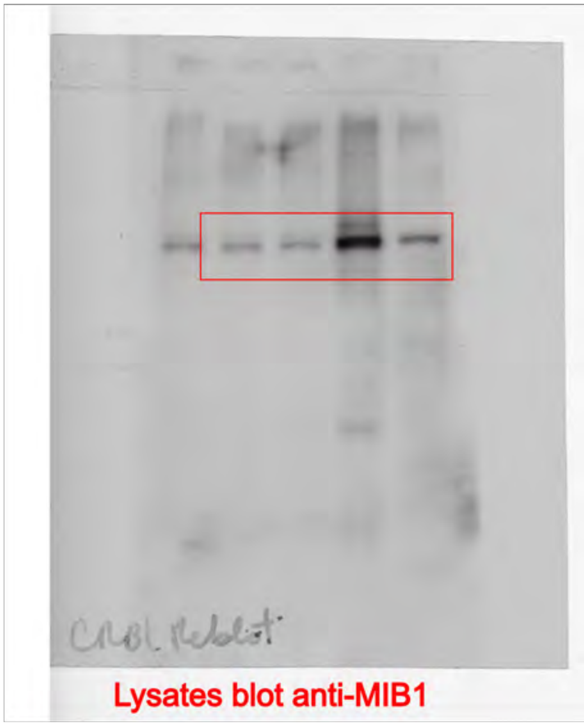

Supplement: Supplementary file 2 — Supplementary information [file 41598_2019_48902_MOESM2_ESM.pdf]
